# PIEZO1 transduces mechanical itch in mice

**DOI:** 10.1038/s41586-022-04860-5

**Published:** 2022-06-22

**Authors:** Rose Z. Hill, Meaghan C. Loud, Adrienne E. Dubin, Brooke Peet, Ardem Patapoutian

**Affiliations:** 1 Howard Hughes Medical Institute, Department of Neuroscience, Dorris Neuroscience Center, The Scripps Research Institute, La Jolla, CA USA; 2 Department of Neuroscience, Dorris Neuroscience Center, The Scripps Research Institute, La Jolla, CA USA

**Keywords:** Somatic system, Neuronal physiology, Ion channels in the nervous system, Somatosensory system, Neurophysiology

## Abstract

Itch triggers scratching, a behavioural defence mechanism that aids in the removal of harmful irritants and parasites^1^. Chemical itch is triggered by many endogenous and exogenous cues, such as pro-inflammatory histamine, which is released during an allergic reaction^1^. Mechanical itch can be triggered by light sensations such as wool fibres or a crawling insect^2^. In contrast to chemical itch pathways, which have been extensively studied, the mechanisms that underlie the transduction of mechanical itch are largely unknown. Here we show that the mechanically activated ion channel PIEZO1 (ref. ^3^) is selectively expressed by itch-specific sensory neurons and is required for their mechanically activated currents. Loss of PIEZO1 function in peripheral neurons greatly reduces mechanically evoked scratching behaviours and both acute and chronic itch-evoked sensitization. Finally, mice expressing a gain-of-function *Piezo1* allele^4^ exhibit enhanced mechanical itch behaviours. Our studies reveal the polymodal nature of itch sensory neurons and identify a role for PIEZO1 in the sensation of itch.

## Abstract (web summary)

Experiments in mice show that the mechanically activated ion channel PIEZO1 is expressed in itch-specific sensory neurons and has a role in transducing mechanical itch.

## Main

Discrete subsets of somatosensory neurons within the dorsal root ganglion (DRG) and trigeminal ganglion selectively drive itch behaviours in mice^5,6^. These pruriceptors are thought to be chemosensory rather than mechanosensory and, as such, are selectively implicated in chemical itch^1^. Chemical pruriceptors are marked by the expression of the neuropeptide genes somatostatin (*Sst*) and natriuretic polypeptide precursor B (*Nppb*), or the MAS-related G-protein coupled receptor A3 (*Mrgpra3*), along with other marker genes identified from single-cell RNA sequencing (RNA-seq) studies^7–9^. Previous work elucidated chemical itch transduction pathways in these neurons^5,10–13^. By contrast, the molecular and cellular basis of mechanical itch in the periphery is relatively unknown, although its circuitry is well described in the spinal cord. For example, mechanical itch can be triggered by the activation of Toll-like receptor 5-positive Aβ-low threshold mechanoreceptors (LTMRs) and the engagement of urocortin 3- and neuropeptide Y receptor 1-positive excitatory spinal pathways, and/or by the inhibition of spinal neuropeptide Y-positive inhibitory interneurons that receive LTMR input^14–16^. Furthermore, age-dependent loss of Merkel cell–neurite complexes enhances mechanical itch, presumably through the loss of LTMR-dependent inhibition of itch spinal circuitry^17^. The contributions of other somatosensory neurons, including chemical pruriceptors, to mechanical itch and itch sensitization are unknown.

## Itch neurons express functional PIEZO1

**Fig. 1:**
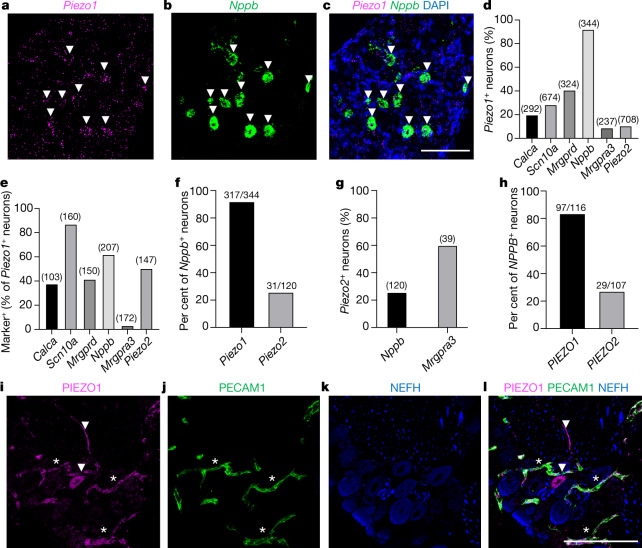
PIEZO1 is expressed in mouse and human putative itch receptors. **a**–**c**, Representative images (five images from two mice) of sectioned mouse DRG smFISH for *Piezo1* (**a**), *Nppb* (**b**) and merged with DAPI (**c**). White arrowheads indicate *Piezo1*^*+*^ or *Nppb*^+^ cells. **d**, Quantification of mouse DRG smFISH images showing the percentage of cells expressing a given marker (bar labels) that were co-labelled with *Piezo1* transcript. The number of analysed neurons is indicated above the bar (four to six images per marker from two mice). **e**, Data from **d** presented as the percentage of *Piezo1*^+^ neurons that co-express a given marker. **f**, Percentage of *Nppb*^+^ neurons expressing *Piezo1* or *Piezo2*. **g**, Comparison of *Piezo2* expression in *Nppb*^+^ versus *Mrgpra3*^*+*^ neurons. **h**, Quantification of human smFISH images (seven to eight images per marker from one donor; see Extended Data Fig. 4a,b). **i**–**l**, Representative images of sectioned mouse DRGs labelled with antibodies against tdTomato (images show PIEZO1 (**i**), PECAM1 (**j**), neurofilament H (NEFH; **k**) and the merged image (**l**)). Asterisks indicate blood vessels and arrowheads indicate a PIEZO1^+^ neuron and nerve fibre. The experiment was repeated one additional time. All images are presented as maximum intensity *z*-projections of confocal images. Scale bars, 100 µm.

**Extended Data Fig. 1:**
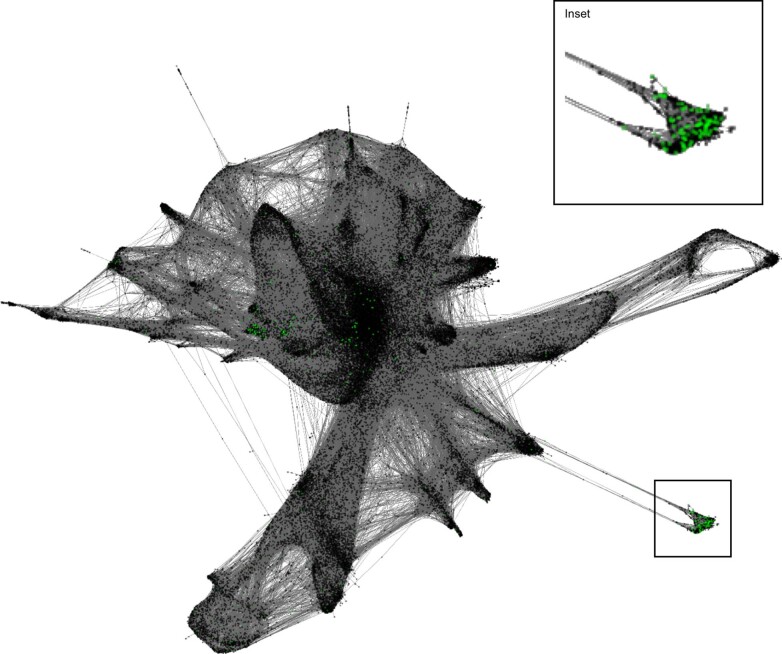
Expression of *Piezo1* in pruriceptive sensory neurons. Single-cell RNA-seq data from a previous report^8^ presented as a force-directed layout showing expression of *Piezo1* in the cluster containing *Sst/Nppb*^+^ DRG neurons (inset). Downloaded from: https://kleintools.hms.harvard.edu/tools/springViewer_1_6_dev.html?datasets/Sharma2019/all.

**Extended Data Fig. 2:**
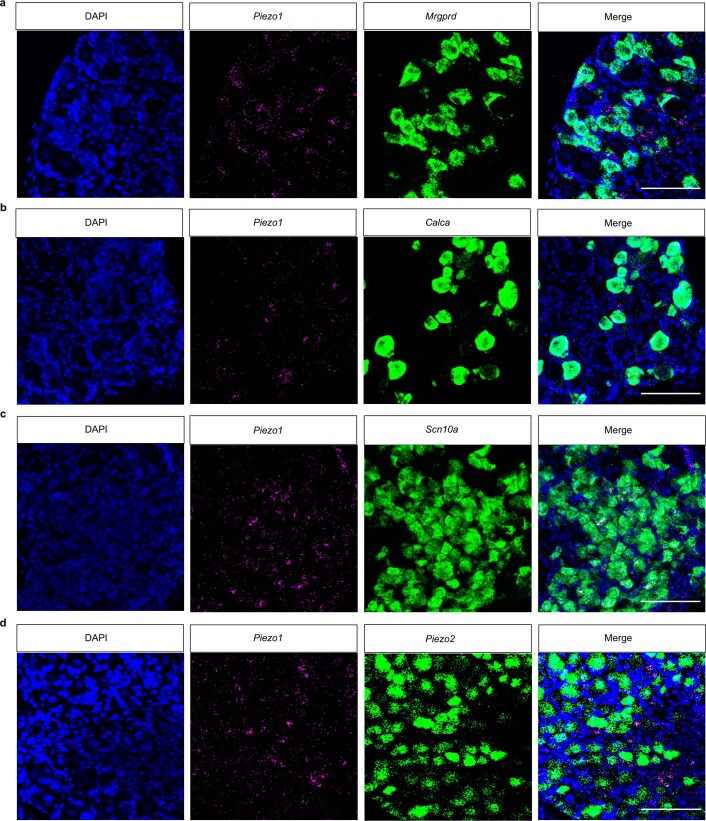
Expression of Piezo genes in nociceptive sensory neurons. **a**–**d**, Representative images (see Fig. 1d, e, 4–6 images per marker from 2 mice) of sectioned mouse DRG processed for smFISH of *Piezo1* (**a**–**d**), *Mrgprd* (**a**), *Calca* (**b**), *Scn10a* (**c**), and *Piezo2* (**d**) with DAPI stain and merged image. Scale = 100 µm.

**Extended Data Fig. 3:**
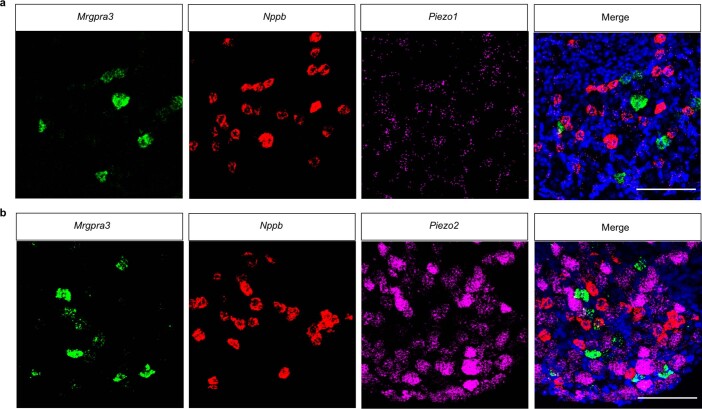
Expression of Piezo genes in pruriceptive sensory neurons. **a**,**b**, Representative images (see Fig. 1d–g, 4–6 images per marker from 2 mice) of sectioned mouse DRG processed for smFISH of *Mrgpra3* (**a**, **b**), *Nppb* (**a**, **b**), *Piezo1* (**a**), and *Piezo2* (**b**) with DAPI stain in the merged image. Scale = 100 µm.

**Extended Data Fig. 4:**
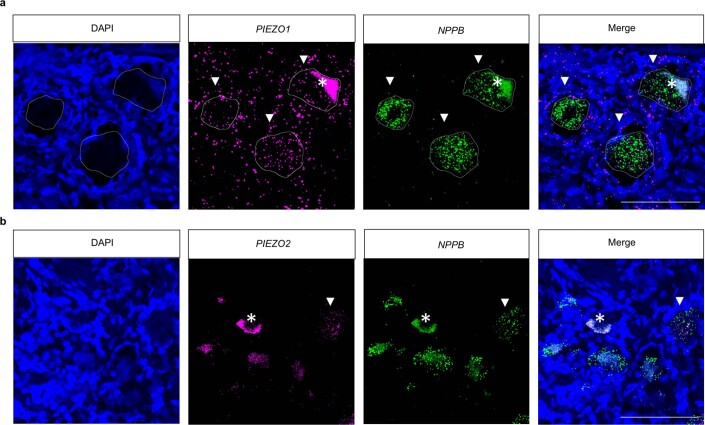
Expression of PIEZO genes in human DRG. **a**, Representative image (see Fig. 1h, 7-8 images per marker from 1 donor) of sectioned T1 human DRG smFISH for *PIEZO1* (magenta) and *NPPB* (green), and merged image with DAPI (blue). **b**, Representative image (see Fig. 1h) of sectioned T1 human DRG smFISH for *PIEZO2* (magenta) and *NPPB* (green), and merged image with DAPI (blue). Asterisks denote lipofuscin signal and arrowheads denote *PIEZO*^*+*^*NPPB*^*+*^ cells. Scale = 100 µm.

**Extended Data Fig. 5:**
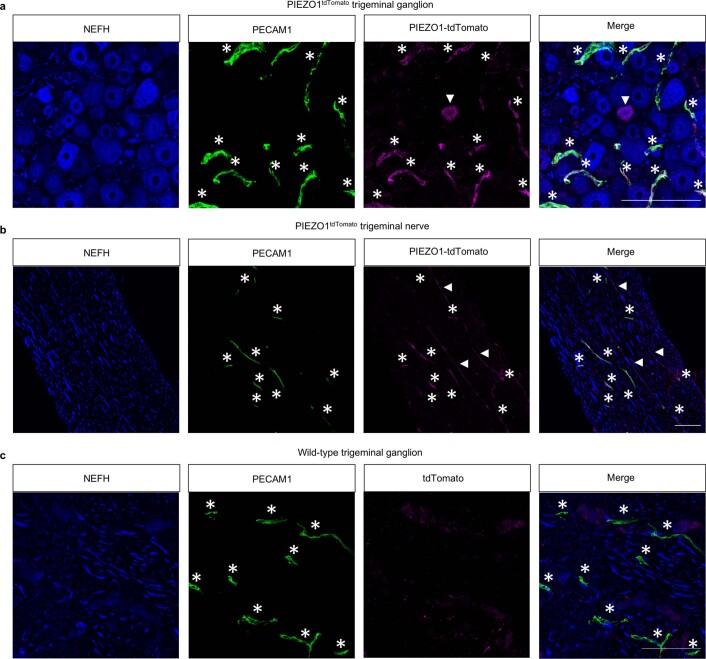
Expression of PIEZO1 in somatosensory neurons. **a**, Sectioned PIEZO1^tdTomato^ mouse trigeminal ganglia labelled with antibodies against NEFH, PECAM1, and tdTomato (PIEZO1), and merged image. **b**, Sectioned PIEZO1^tdTomato^ mouse trigeminal nerve labelled with antibodies against NEFH, PECAM1, and tdTomato (PIEZO1), and merged image. **c**, Sectioned wild-type mouse trigeminal ganglia labelled with antibodies against NEFH, PECAM1, and tdTomato, and merged image. Asterisks indicate PECAM1^+^ blood vessels and arrowheads indicate PIEZO1^+^ neurons and nerve fibres. All images are presented as maximum intensity z-projections of confocal images; scale bars = 100 µm. Experiment was repeated one additional time.

Early work on the expression of Piezo genes unequivocally demonstrated that *Piezo2* transcript is present at high levels in somatosensory neurons and suggested that *Piezo1* was expressed at only background levels^3,18^. In response to the publication of several single-cell RNA-seq datasets reporting low but detectable expression of *Piezo1* transcript in mouse DRG neurons^7,8,19^ (Extended Data Fig. 1), we revisited our previous experiments using single-molecule fluorescence in situ hybridization (smFISH) to more thoroughly characterize the expression of *Piezo1* in sensory neurons. Notably, we observed that *Piezo1* is expressed in 92% of *Nppb*^+^ DRG neurons (Fig. 1a–f and Extended Data Fig. 2a–d), and in non-neuronal cells that are likely to comprise vascular endothelium^20^. This specific expression pattern suggested that PIEZO1 has a role in itch. We also observed the expression of *Piezo1* in a subset of MAS-related G-protein coupled receptor D (*Mrgprd*^+^) neurons (Extended Data Fig. 2a), which have previously been implicated in mechanical pain and in chemical itch evoked by the compound β-alanine^21,22^; however, we did not observe *Piezo1* in the *Mrgpra3*^+^ chloroquine-sensitive chemical itch neurons (Fig. 1d–f and Extended Data Fig. 3a). By contrast, *Piezo2* was expressed in a smaller percentage of *Nppb*^*+*^ neurons (Fig. 1d–f and Extended Data Fig. 3b). Substantial differences have been identified between human and mouse DRG neurons^23,24^ and the role—if any—of *NPPB* in human itch transmission is unknown. smFISH of sections from a human DRG revealed *PIEZO1* transcript in 83.6% of *NPPB*^*+*^ neurons (Fig. 1h and Extended Data Fig. 4a,b), suggestive of a conserved pattern of expression. Using a mouse line that expresses a PIEZO1^tdTomato^C-terminal fusion protein^25^, we observed robust expression of PIEZO1^tdTomato^ in platelet endothelial cell adhesion molecule 1 (PECAM1^+^) vascular endothelial cells of the DRG and trigeminal ganglion capillaries (as expected)^20^, as well as within a subset of neuronal cell bodies and nerve fibres, consistent with the expression of PIEZO1 protein in mouse somatosensory neurons (Fig. 1i–l and Extended Data Fig. 5a–c).

**Fig. 2:**
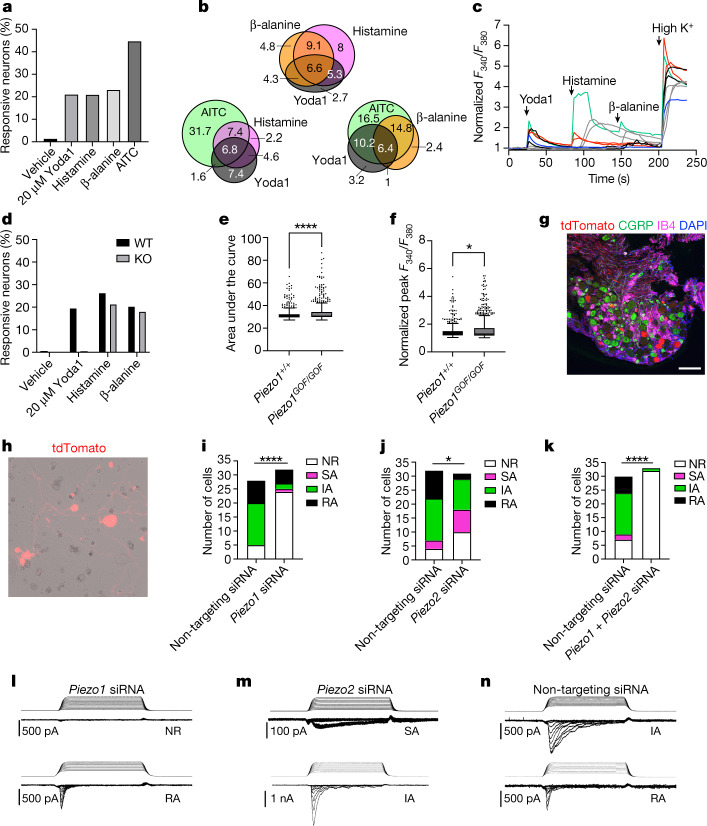
PIEZO1 is functionally expressed in a subset of putative itch neurons. **a**, Percentage of wild-type neurons responding to compounds (2,682 neurons from 3 mice). AITC, allyl isothiocyanate. **b**, Venn diagrams of response overlap from combined wild-type data in **a** and **d**, indicating the percentage of total neurons. **c**, Traces of representative calcium signals from **b**. Arrowheads indicate compound addition. **d**, Percentage of neurons responding in *Piezo1*^ *fl/fl*^*;Pirt*^ *Cre−/−*^ (wild type; WT) versus *Piezo1*^ *fl/fl*^*;Pirt*^ *Cre+/−*^ (KO) neurons (1,883 WT and 2,218 KO neurons from 2 mice per genotype). **e**, Area under the curve of *Piezo1*^*+/+*^ versus *Piezo1*^*GOF/GOF*^ responses to 20 µM Yoda1 (Mann–Whitney: ****P < 0.0001, *U* = 102,979; *n* = 347 +/+ and 711 GOF/GOF neurons from 2 mice per genotype). **f**, Peak normalized *F*_340_/*F*_380_ ratio of *Piezo1*^*+/+*^ versus *Piezo1*^*GOF/GOF*^ mice from data in **e** (Mann–Whitney: *P = 0.0142, *U* = 121,046; *n* = 347 +/+ and 768 GOF/GOF neurons from 2 mice). In **e**,**f**, the centre line denotes the median, the boxes are the 25th and 75th percentiles and the whiskers indicate 1.5 times the interquartile range. **g**, Representative immunohistochemistry (IHC) section (of three sections from two mice) of *Ai9* ^*fl/fl*^*;Sst*^*Cre+/*−^ DRG neurons showing native tdTomato (expressed in SST^+^ cells) with the indicated markers (scale bar, 100 µm). **h**, Dissociated *Ai9* ^*fl/fl*^*;Sst*^*Cre+/*−^ DRG neurons. **i**–**k**, Summary of mechanically activated (MA) current inactivation kinetics in whole-cell poke experiments after nucleofection of *Ai9*^ *fl/fl*^*;Sst*^*Cre+/*^^−^ DRG neurons with the indicated siRNA mix against non-targeting control siRNA or against *Piezo1* (**i**; ****P < 0.0001, *χ*^2^ = 23.92, degrees of freedom (df) = 3; *n* = 28 control and 32 *Piezo1* siRNA cells), *Piezo2* (**j**; *P = 0.0130, *χ*^2^ = 10.78, df = 3; *n* = 32 control and 31 *Piezo2* siRNA cells) and *Piezo1* + *Piezo2* (**k**; ****P < 0.0001, *χ*^2^ = 36.21, df = 3; *n* = 30 control and 33 *Piezo1* + *Piezo2* siRNA cells). Chi-squared (*χ*^2^) tests were performed. IA, intermediately adapting; NR, non-responsive; RA, rapidly adapting; SA, slowly adapting. **l**–**n**, Representative 150-ms indentation traces (top) and MA currents (bottom) from **i**–**j** with nucleofection of indicated siRNA (from two mice each). All statistical tests are two-tailed where applicable. *n* indicates biological replicates (cells). Source data

We next sought to establish whether PIEZO1 is functional in somatosensory neurons. Unlike PIEZO2, which to date has no known chemical activators, PIEZO1 can be activated in vitro and in vivo using the small molecule Yoda1, which sensitizes PIEZO1 currents elicited by mechanical stimuli and triggers calcium influx on its own^26^. To assess the effects of Yoda1 on sensory neuron physiology, we turned to ratiometric calcium imaging in dissociated cultured mouse DRG neurons. We observed that Yoda1 triggered calcium transients in approximately 20% of cultured DRG neurons (Fig. 2a) and most of these cells were responsive to the itch compounds β-alanine (a MRGPRD agonist) and/or histamine, which activates a broad subset of TRPV1^+^ somatosensory neurons^27^ including *Nppb*^+^ neurons^5,11^ (Fig. 2b,c). Yoda1-dependent calcium transients were lost in neurons from *Piezo1*^*fl/fl*^*;Pirt-Cre*^*+/−*^ mice, which targets the vast majority of peripheral sensory neurons^28^ (Fig. 2d). Conversely, we tested whether neurons from mice expressing a gain-of-function (GOF) *Piezo1* allele (PIEZO1^GOF^) equivalent to a human hereditary xerocytosis mutation^4^ might exhibit enhanced Yoda1 responses. We observed modest increases in the area under the curve and the peak amplitude of Yoda1 calcium transients in PIEZO1^GOF^ DRG neurons (Fig. 2e,f).

## SST^+^ neurons have PIEZO1-dependent currents

**Extended Data Fig. 6:**
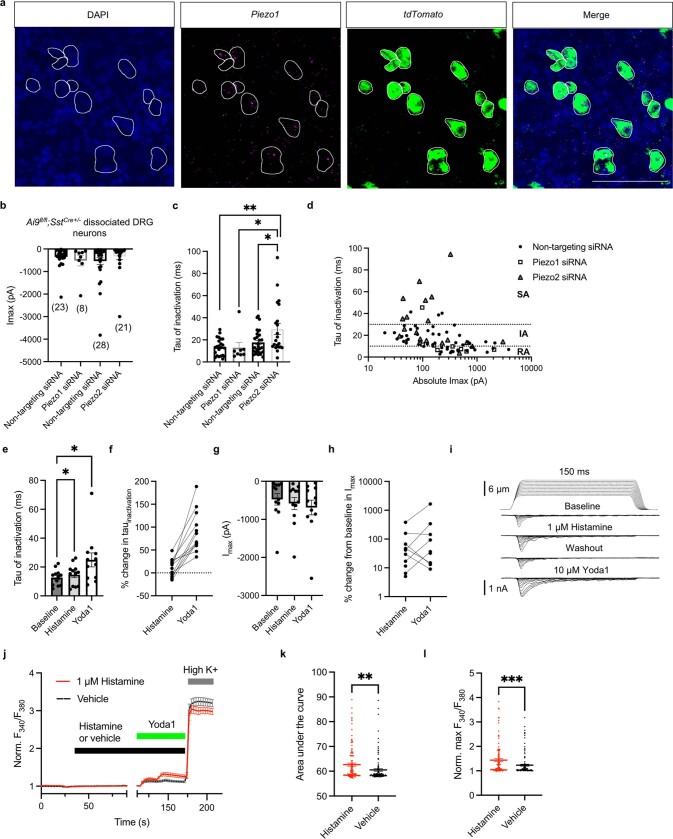
Additional Ca^2+^ imaging and electrophysiology for tdTomato^+^ DRG neurons. **a**, Representative smFISH images (from 2 mice) of *Ai9*^*fl/fl*^; *Sst*^*Cre+/-*^ DRG for *Piezo1* (magenta) and *tdTomato* (green) with DAPI and merged image. Scale = 100 µm. **b**, I_max_ of MA currents in neurons nucleofected with the indicated siRNA pools. Cells that exhibited no MA currents were not included for analysis (One-way ANOVA: *p* = 0.6616; F(3, 76) = 0.5321). Number of responding cells is indicated from 2 mice per condition. **c**, τ_inactivation_ of MA currents from **b** (One-way ANOVA: **p = 0.0021; F(3, 76) = 5.352; Tukey’s (left to right): **p = 0.0026; *p = 0.0346; *p = 0.0270). **d**, I_max_ vs. τ_inactivation_ of MA currents from **b** and **c**, with non-targeting siRNA *n* (black circles) pooled between *Piezo1* (white squares) and *Piezo2* (grey triangles) knockdown experiments. Dotted lines denote limits for SA, IA, and RA current categories. **e**, τ_inactivation_ of MA currents in neurons before (baseline), during 100 µM histamine treatment, washout, and with 10 µM Yoda1 treatment (repeated measures ANOVA: **p = 0.0077; F(2,11) = 10.11; Dunnett’s: *p_histamine_ = 0.0281; *p_Yoda1_ = 0.0115). **f**, data in **e** presented as the % change in τ_inactivation_ compared to baseline (0%). **g**, I_max_ of MA currents from **e** (repeated measures ANOVA: *p* = 0.0714; F(2,11) = 3.737). **h**, data in **g** represented as % change in I_max_ compared to baseline (0%). **e**–**h** are from *n* = 12 cells from 2 mice. **i**, representative traces from experiments in **e**–**h**, with indentation trace shown at top. **j**, Normalized Fura2 ratio plotted over time with addition of Yoda1 following 5 min pre-incubation in 1 µM histamine or vehicle. Some neurons had a delayed response to Yoda1 which is indicated in the biphasic curves for histamine and vehicle. Broken axis indicates 3 min of elapsed time during incubation. **k**, area under the curve for calcium imaging data in **j** (Mann–Whitney: **p = 0.0021, U = 5596). **l**, maximum normalized Fura2 ratio for calcium imaging data in **j** (Mann–Whitney: ***p = 0.0005, U = 5384). **j**–**l** are from *n* = 120 histamine and 121 vehicle neurons from 2 mice. Error bars represent mean ± s.e.m. of *n* biological replicates (cells) and statistical tests are two-tailed where applicable. Source data

We then investigated whether PIEZO1-expressing somatosensory neurons are inherently mechanosensitive. Although chemical pruriceptors are generally believed to be mechanically insensitive, a handful of studies suggest that they may exhibit mechanosensitivity under certain conditions^29^. As *Piezo1* is expressed in only a subset of *Mrgprd*^+^ sensory neurons, we reasoned that a phenotype driven by the perturbation of *Piezo1* expression in these cells might be missed. By contrast, *Piezo1* was expressed in the vast majority of *Nppb*^+^ neurons, and these cells are amenable to genetic targeting with somatostatin^Cre^ (*Sst*^*Cre*^; Fig. 2g), as *Sst* is expressed in virtually all mouse *Nppb*^+^ neurons^5,19^. We validated the expression of *Piezo1* within *Ai9*^ *fl/fl*^*;Sst* ^*Cre+/−*^ *tdTomato*^+^ cells by RNAscope and found that 73.9% of *tdTomato*^+^ cells co-expressed *Piezo1* (Extended Data Fig. 6a). We performed whole-cell electrophysiology to examine mechanically activated currents in *tdTomato*^+^ cultured DRG neurons from *Ai9*^ *fl/fl*^*;Sst*^ *Cre+/−*^ mice (Fig. 2h). To perturb the function of mechanically activated channels, we nucleofected pooled small interfering RNAs (siRNAs) against *Piezo1* and/or *Piezo2* or non-targeting control siRNA^3,18,30^. In all experiments, we co-nucleofected a plasmid to drive the cytosolic expression of green fluorescent protein (GFP) and recorded from GFP^+^tdTomato^+^ cells. In 45 out of 74 mechanosensitive cells nucleofected with the non-targeting siRNA (*n* = 90 recordings), we observed intermediately adapting currents (10 ms < *τ*_inactivation_ (the time constant of current inactivation) < 30 ms) in response to controlled indentation with a blunt glass probe, with 24 out of 74 mechanosensitive cells exhibiting rapidly adapting currents (*τ*_inactivation_ ≤ 10 ms; Figs. 2i–k,n and Extended Data Fig. 6b–d). With *Piezo1* siRNA nucleofection, intermediately adapting currents were largely lost, and rapidly adapting currents were retained (Fig. 2i,l,n and Extended Data Fig. 6c). This is consistent with published data that show that PIEZO1-dependent currents have a larger *τ*_inactivation_ than PIEZO2-dependent currents in endogenous and heterologous expression systems^3,31^. By contrast, after nucleofection of *Piezo2* siRNA, the rapidly adapting responses were lost and the intermediately adapting responses remained (Fig. 2j,m,n and Extended Data Fig. 6b–d). This is consistent with the role of PIEZO2 in mediating nearly all rapidly adapting currents in DRG neurons^18^. Of note, some slowly adapting currents (*τ*_inactivation_ ≥ 30 ms) were observed after the knockdown of *Piezo2*, suggestive of the unmasking of slowly adapting responses after loss of the rapidly adapting channel, as slowly adapting currents were infrequently observed in control cells. With simultaneous knockdown of both *Piezo1* and *Piezo2*, 32 out of 33 cells were unresponsive to mechanical stimuli (Fig. 2k). Thus, PIEZO1 is the predominant mediator of mechanically activated currents in SST^+^ DRG neurons, with PIEZO2 contributing to rapidly adapting currents.

Hypersensitivity to mechanical itch (alloknesis) arises after the injection of histamine into the skin of mice and humans^32,33^. Histamine-responsive DRG neurons are primarily composed of dedicated TRPV1^+^ itch receptors, including *Nppb*^+^ neurons as well as *Mrgpra3*^*+*^ neurons^11^. We investigated whether histamine could directly sensitize PIEZO1-dependent mechanically activated currents^26^ in *Ai9*^*fl/fl*^*;Sst*^*Cre+/−*^ DRG neurons. A five-minute exposure to 100 µM histamine caused a small increase in the *τ*_inactivation_ of presumptive PIEZO1-dependent mechanically activated currents that were also sensitized by 10 µM Yoda1 (Extended Data Fig. 6e,f,i). Neither Yoda1 nor histamine significantly altered the maximal current, *I*_max_ (Extended Data Fig. 6g,h). Incubation in a sub-threshold concentration of histamine^34^ (1 µM), which is insufficient to drive calcium influx, similarly enhanced Yoda1-dependent calcium influx in tdTomato^+^ DRG neurons (Extended Data Fig. 6j–l). Aside from direct effects on PIEZO1-dependent mechanically activated currents, histamine and other pruritogens may drive itch sensitization through cell-autonomous or -non-autonomous mechanisms, the latter analogous to the sensitization of LTMR circuitry that is observed in allodynia^35^. These findings suggest a potential role for PIEZO1-dependent mechanotransduction in alloknesis.

## PIEZO1 mediates mechanical itch behaviours

**Fig. 3:**
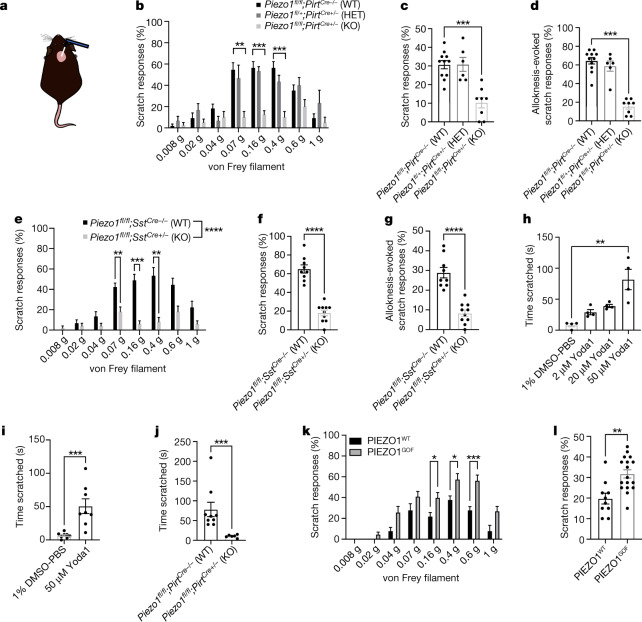
Neuronal PIEZO1 is required for mechanically evoked scratching and histamine alloknesis in mice. **a**, Illustration of the nape model of mechanical itch. **b**, Mechanical itch model in *Piezo1*^*fl/fl*^ or *Piezo1* ^fl^^/+^;*Pirt*^*Cre−/*^^*−*^ (WT; *n* = 11), *Piezo1* ^*fl/+*^*;Pirt*^*Cre+/*^^*−*^ (heterozygous (HET); *n* = 6) and *Piezo1* ^*fl/fl*^*;Pirt*^*Cre+/*^^*−*^ (KO; *n* = 8) mice (two-way ANOVA: ****P_genotype_ < 0.0001, *F*(2, 22) = 17.88; Sidak’s *P*_adjusted_: **P_0.07g_ = 0.0017, ***P_0.16g_ = 0.0003, ***P_0.4g_ = 0.0005). **c**, Cumulative per cent scratch responses from **b** (Kruskal–Wallis: ***P = 0.0007, *χ*^2^ = 14.52; Dunn’s ***P_adjusted_ = 0.0008). **d**, Histamine alloknesis (Kruskal–Wallis: ***P = 0.0003, *χ*^2^ = 16.43; Dunn’s: ***P_adjusted_ = 0.0001) from mice in **b**. Data in **b**–**d** are from three experiments. **e**, Mechanical itch model in *Piezo1*^*fl/fl*^;*Sst*^*Cre*^^*−/−*^ (WT; *n* = 8) and *Piezo1* ^*fl/fl*^*;Sst*^ *Cre+/*^^*−*^ (KO; *n* = 10) mice (two-way ANOVA: ****P_genotype_ < 0.0001, *F*(1, 17) = 44.87; Sidak’s *P*_adjusted_: **P_0.07g_ = 0.0086, ***P_0.16g_ = 0.0002, **P_0.4g_ = 0.0027). **f**, Cumulative per cent scratch responses from **e** (Mann–Whitney: ****P < 0.0001, *U* = 0). **g**, Histamine alloknesis (Mann–Whitney: ****P < 0.0001, *U* = 0) from mice in **e**. Data in **e**–**g** are from two experiments. **h**, Cheek model of Yoda1-evoked itch (Kruskal–Wallis: ****P < 0.0001, *χ*^2^ = 12.88; Dunn’s: **P_adjusted_ = 0.0014; *n* = 4 mice from 1 experiment). No wiping was observed. **i**, Nape model of Yoda1-evoked itch (Mann–Whitney: ***P = 0.0003, *U* = 1; *n* = 8 mice from 1 experiment). **j**, Nape model of itch (50 µM Yoda1) in *Piezo1*^ *fl/fl*^ or *Piezo1* ^*fl/+*^*;Pirt*^ *Cre−/−*^ (WT) and *Piezo1* ^*fl/fl*^;*Pirt*^*Cre+/−*^ (KO) mice (Mann–Whitney: ***P = 0.0004, *U* = 0; *n* = 9 WT and 6 KO mice from 2 experiments). **k**, Mechanical itch model in *Piezo1*^*+/+*^ (PIEZO1^WT^; *n* = 9) and *Piezo1*^*GOF/GOF*^ or *Piezo1*^*GOF/+*^ (PIEZO1^GOF^; *n* = 17) mice (two-way ANOVA: ****P_genotype_ < 0.0001, *F*(1, 25) = 24.16; Sidak’s *P*_adjusted_: *P_0.16g_ = 0.0493, *P_0.4g_ = 0.0441, ***P_0.6g_ = 0.0008). **l**, Cumulative per cent scratch responses from **k** (Mann–Whitney: **P = 0.0013, *U* = 14). Error bars represent mean ± s.e.m. of *n* biological replicates (mice) and statistical tests are two-tailed where applicable. Data in **k**–**l** are from three experiments. Source data

**Extended Data Fig. 7:**
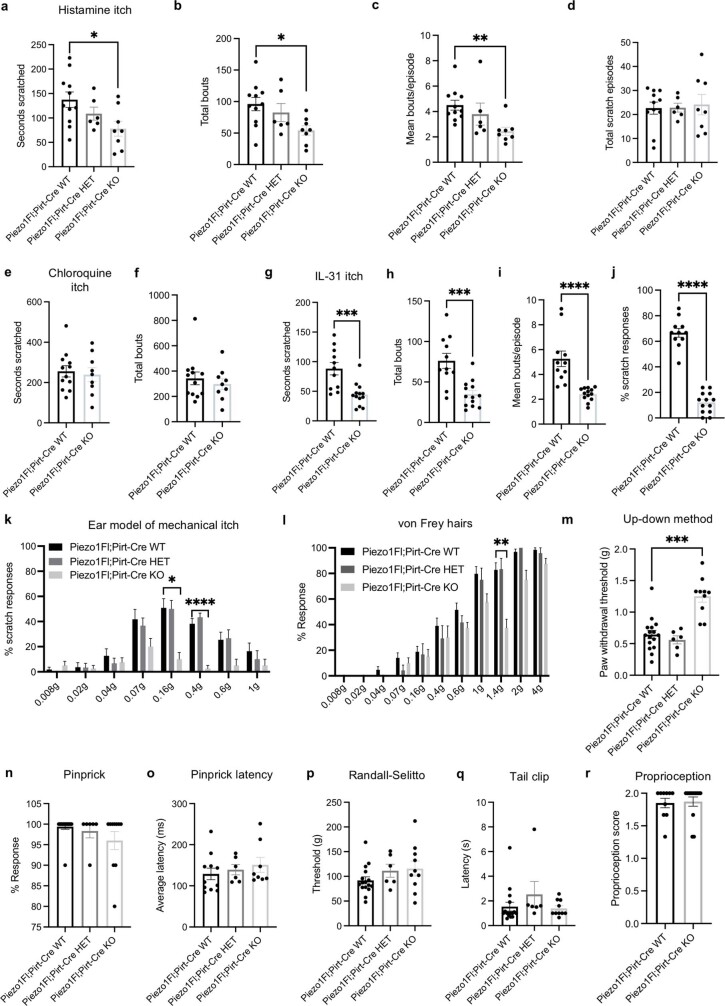
Additional somatosensory behaviours in *Piezo1* ^*fl/fl*^;*Pirt*^*Cre*^ mice. **a**, Histamine-evoked itch (Kruskal–Wallis: *p = 0.0386; χ^2^ = 6.510; Dunn’s: *p_adjusted_ = 0.0216). **b**, Total scratch bouts following histamine (Kruskal–Wallis: *p = 0.0296; χ^2^ = 7.043; Dunn’s: *p = 0.0165). **c**, Mean bouts per episode from **a** (Kruskal–Wallis: **p = 0.0047; χ^2^ = 10.73; Dunn’s: **p_adjusted_ = 0.0021). **d**, Scratch episodes following histamine (Kruskal–Wallis: *p* = 0.9401; χ^2^ = 0.1234). **a**–**d** are from *n* =11 WT, *n* = 6 HET, and *n* = 8 KO mice from 3 experiments. **e**, Chloroquine-evoked itch (Mann–Whitney: *p* = 0.8078; U = 50, n = 12, 9). **f**, Total scratch bouts following chloroquine (Mann–Whitney: *p* = 0.6511; U = 47). **e**-**f** are from *n* = 12 WT and *n* = 9 KO mice from 2 experiments. **g**, IL-31-evoked itch (Mann–Whitney: ***p = 0.0008; U = 16.50, n = 11, 13). **h**, Total scratch bouts following IL-31 (Mann–Whitney: ***p = 0.0009; U = 17). **i**, Mean bouts per episode from **g** (Mann–Whitney: ****p < 0.0001; U = 0.5). **j**, IL-31-evoked alloknesis (Mann–Whitney: ****p < 0.0001; U = 0). **g**-**i** are from *n* = 11 WT and *n* = 13 KO mice from 2 experiments. **k**, Ear model of mechanical itch (Two-way ANOVA: ***p_genotype_ = 0.0002; F(2, 22) = 12.93; Sidak’s *p*_adjusted_: *p_0.16g_ = 0.0214; ****p_0.4g_ < 0.0001, *n* = 11 WT, *n* = 6 HET, and *n* = 8 KO mice from 3 experiments). **l**, % withdrawal response (Two-way ANOVA: ***p_genotype_ = 0.0007; F(2, 29) = 9.524; Dunn’s **p_adjusted_ = 0.0014). **m**, 50% withdrawal von Frey threshold (Kruskal–Wallis: ***p = 0.0002; χ^2^ = 16.71; Dunn’s: ***p = 0.0007). **n**, Pinprick response (Kruskal–Wallis: *p* = 0.2568; χ^2^ = 2.719). **o**, Pinprick latency (Kruskal–Wallis: *p* = 0.3500; χ^2^ = 2.100, N = 11 WT, N = 6 HET, and N = 8 KO mice from 3 experiments). **p**, Randall–Selitto (Kruskal–Wallis: *p* = 0.2225; χ^2^ = 3.006). **q**, Tail clip (Kruskal–Wallis: *p* = 0.2505, χ^2^ = 2.769). Data in **l**–**n** and **p**,**q** are from *n* = 16 WT, *n* = 6 HET, and *n* = 10 KO mice from 4 experiments. **r**, Proprioception scores (Mann–Whitney: *p* = 0.6430, U = 57, *n* = 10, 13 mice from 1 experiment). Error bars represent mean ± s.e.m. of *n* biological replicates (mice) and statistical tests are two-tailed where applicable. Source data

In order to ascertain the role of sensory neuronal PIEZO1 in mechanical itch and alloknesis, we depleted PIEZO1 from peripheral sensory neurons using the *Pirt*^*Cre*^ driver in a *Piezo1*^*flox*^ mouse (*Piezo1* ^*fl/fl*^*;Pirt*^*Cre+/−*^) (ref. ^28^) and tested the resulting mice in models of acute chemical and mechanical itch. We chose this pan-sensory neuronal Cre driver to delete PIEZO1 from all peripheral neurons. In the nape model of mechanical itch^14,15,32^ (Fig. 3a), we observed a profound decrease in mechanically evoked scratching in conditional knockout (KO) mice, but not in heterozygous or wild-type littermate controls (Fig. 3b,c). Moreover, although KO mice were capable of scratching in response to histamine injection into the nape, KO mice did not exhibit robust alloknesis to stimulation with the 0.04 g von Frey filament after the cessation of histamine-evoked scratching^14,32^ (Fig. 3d and Extended Data Fig. 7a–d). We chose the 0.04 g filament for alloknesis assays because it did not evoke substantial scratching in naive wild-type mice (Fig. 3b). Although KO mice did scratch in response to acute histamine injection, we observed a small but significant reduction that was most apparent in a reduced number of scratching bouts per scratching episode^36^ (Extended Data Fig. 7b–d). To further investigate this finding, we also tested the ability of KO mice to scratch in response to the injection of chloroquine, which activates MRGPRA3^+^ itch receptors^12^ (that do not express *Piezo1*) or that of of interleukin-31 (IL-31), which activates *Nppb*^+^ neurons that co-express *Il31ra (*ref. ^7^). Chloroquine-evoked itch (Extended Data Fig. 7e,f) was normal in KO mice, whereas IL-31-evoked itch and alloknesis were decreased similarly to histamine (Extended Data Fig. 7g–j). These minor deficits in histamine- and IL-31-evoked (but not chloroquine-evoked) chemical itch may reflect a role for PIEZO1-dependent mechanical itch in amplifying scratching behaviours that depend on *Sst*^*+*^*Nppb*^*+*^ neurons. To relate our findings to previous studies of mechanical itch that also tested scratching responses to ear stimulation^14,15^, we also probed the shaved area behind the ears of mice and observed that KO mice had profoundly reduced mechanical itch responses (Extended Data Fig. 7k).

When we examined other mechanosensory behaviours in the KO mice, we observed a small but significant increase in paw withdrawal threshold as measured using the von Frey assay (Extended Data Fig. 7l–m); however, mechanonociceptive reflex behaviours to punctate and blunt stimuli were normal (Extended Data Fig. 7n–q). Proprioceptive behaviours were unaffected (Extended Data Fig. 7r), unlike with the sensory-specific loss of PIEZO2 (ref. ^37^). We postulate that the small effect on mechanical threshold could be due to a potential role for *Piezo1*-expressing *Sst*^*+*^*Nppb*^+^ and/or MRGPRD^+^ neurons in baseline mechanosensitivity—a function suggested previously for these neuronal subpopulations^5,22^. We hypothesized that PIEZO1 acts primarily through the *Sst*^*+*^*Nppb*^+^ subpopulation of itch neurons to promote mechanical itch and itch sensitization, as *Piezo1* was more strongly expressed within this subpopulation than within the *Mrgprd*^+^ cells (Fig. 1), whereas the majority of mechanically activated currents in MRGPRD^+^ cells are dependent on PIEZO2 (ref. ^30^). To this end, we generated *Piezo1*^*fl/fl*^*;Sst*^*Cre+/−*^ mice and observed that they largely phenocopied the *Piezo1*^*fl/fl*^*;Pirt*^*Cre+/−*^ mice with respect to mechanical itch and histamine-evoked alloknesis (Fig. 3e–g). Thus, we conclude that PIEZO1 transduces mechanical itch and alloknesis primarily through *Sst*^*+*^*Nppb*^+^ dedicated itch receptors.

**Extended Data Fig. 8:**
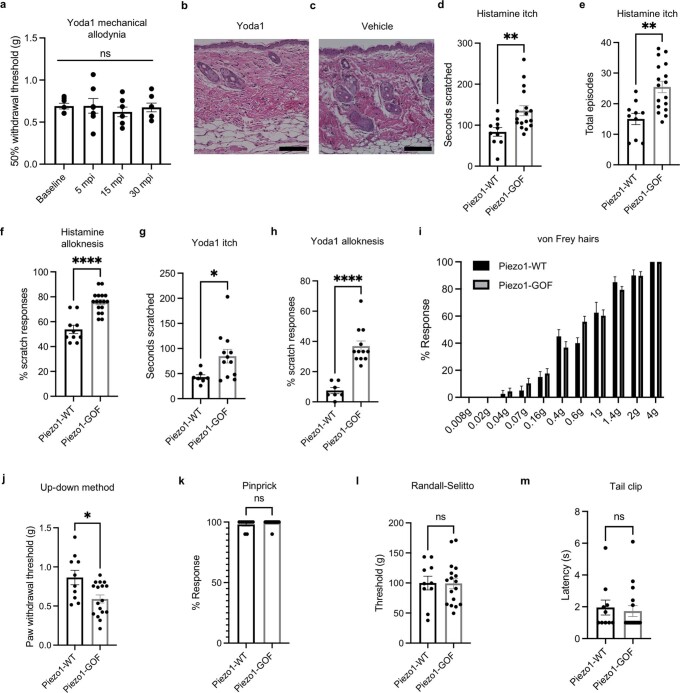
Additional somatosensory behaviours in Yoda1-injected and PIEZO1^GOF^ mice. **a**, 50% withdrawal threshold in wild-type mice measured before (baseline) and after intraplantar injection of Yoda1 at indicated minutes post-injection (mpi) (Friedman: *p* = 0.5433; Friedman statistic = 2.143, *n* = 7 mice from 1 experiment). **b**, Skin section from Yoda1-injected mouse stained with H&E (representative of 5 mice, scale = 100 µm). **c**, Skin section from vehicle-injected mouse stained with H&E (representative of 5 mice, scale = 100 µm). **d**, Histamine-evoked itch (Mann–Whitney: **p = 0.0045; U = 30). **e**, Scratch episodes following histamine in **d** (Mann–Whitney: **p = 0.0018; U = 25.50). **f**, Histamine alloknesis (Mann–Whitney: ****p < 0.0001; U = 10). **d**–**f** are from *n* = 10 WT and *n* = 17 GOF mice from 3 experiments. **g**, Yoda1-evoked itch in *Piezo1*^*+/+*^ and *Piezo1*^*GOF/GOF*^ or *Piezo1*^*GOF/+*^ male and female mice (Mann–Whitney: *p = 0.0180; U = 14.50). **h**, Yoda1 alloknesis (Mann–Whitney: ****p < 0.0001; U = 0). **i**, % withdrawal response (Two-way ANOVA: *p*_genotype_ = 0.6151; F(1, 25) = 0.2592). Data in g,h are from *n* = 7 WT and *n* = 12 GOF mice from 2 experiments. **j**, 50% withdrawal von Frey threshold (Mann–Whitney: *p = 0.0276; U = 41.50). **k**, Pinprick response (Mann–Whitney: *p* = 0.5350; U = 73). **l**, Randall–Selitto (Mann–Whitney: *p* = 0.9411; U = 83). **m**, Tail clip (Mann–Whitney: *p* = 0.5152; U = 73.50). Data in **i**–**m** are from *n* = 10 WT and *n* = 17 GOF mice from 3 experiments. Error bars represent mean ± s.e.m. of *n* biological replicates (mice) and statistical tests are two-tailed where applicable. Source data

We subsequently investigated whether the activation of PIEZO1 can trigger acute itch. We found that injection of Yoda1 selectively induced scratching behaviours in the cheek model, which allows for discrimination between itch-evoked hind limb scratching and pain-evoked forepaw wiping^38^ (Fig. 3h), and induced robust scratching in the nape model of itch (Fig. 3i). No mechanical allodynia was observed after intraplantar injection of Yoda1 (Extended Data Fig. 8a). Notably, *Piezo1*^*fl/fl*^*;Pirt*^*Cre+/*^^*−*^ mice did not exhibit scratching in response to Yoda1, suggestive of a sensory-neuron-specific mechanism by which Yoda1 and PIEZO1 selectively trigger itch and not pain (Fig. 3j). In addition, we observed no overt signs of inflammation 30 min after the injection of Yoda1 into the nape skin (Extended Data Fig. 8b,c), supporting our knockout studies that showed that Yoda1 evokes acute itch primarily through somatosensory neurons rather than through indirect effects on PIEZO1^+^ immune cells or keratinocytes.

To answer the question of whether enhanced PIEZO1 activity modulates mechanical itch in vivo, we took advantage of an existing PIEZO1 gain-of-function mouse model^4,39^. Constitutive PIEZO1^GOF^ mice showed enhanced mechanically evoked scratching behaviours in response to von Frey stimulation of the shaved nape compared to controls (Fig. 3k–l). In addition, PIEZO1^GOF^ mice exhibited increased histamine-evoked alloknesis (Extended Data Fig. 8f). We also observed a slight increase in histamine-evoked itch in PIEZO1^GOF^ mice (Extended Data Fig. 8d,e), implying overall increases in itch-evoked scratching in this mouse line, and consistent with the opposite effect that was observed in *Piezo1*^*fl/fl*^*;Pirt*^*Cre+/−*^ mice. Furthermore, PIEZO1^GOF^ mice exhibited enhanced itch-evoked scratching after the injection of Yoda1 (Extended Data Fig. 8g), and unlike wild-type littermate controls, developed alloknesis after Yoda1 injection (Extended Data Fig. 8h). We speculate that the alloknesis phenotype could be due to enhanced activation of GOF itch receptors by Yoda1, which is administered at a dose limited by the solubility of the molecule^26^. Consistent with the minor increase in hind paw mechanical threshold in the PIEZO1^KO^ mice, we observed no evidence of constitutive allodynia (Extended Data Fig. 8i) and only a slight decrease in the 50% withdrawal threshold of PIEZO1^GOF^ mice (Extended Data Fig. 8j). There was no enhancement of acute mechanonociceptive reflexes to punctate or blunt stimuli (Extended Data Fig. 8k–m). These results demonstrate that enhanced PIEZO1 activity selectively exacerbates mechanical itch and alloknesis in vivo.

**Fig. 4:**
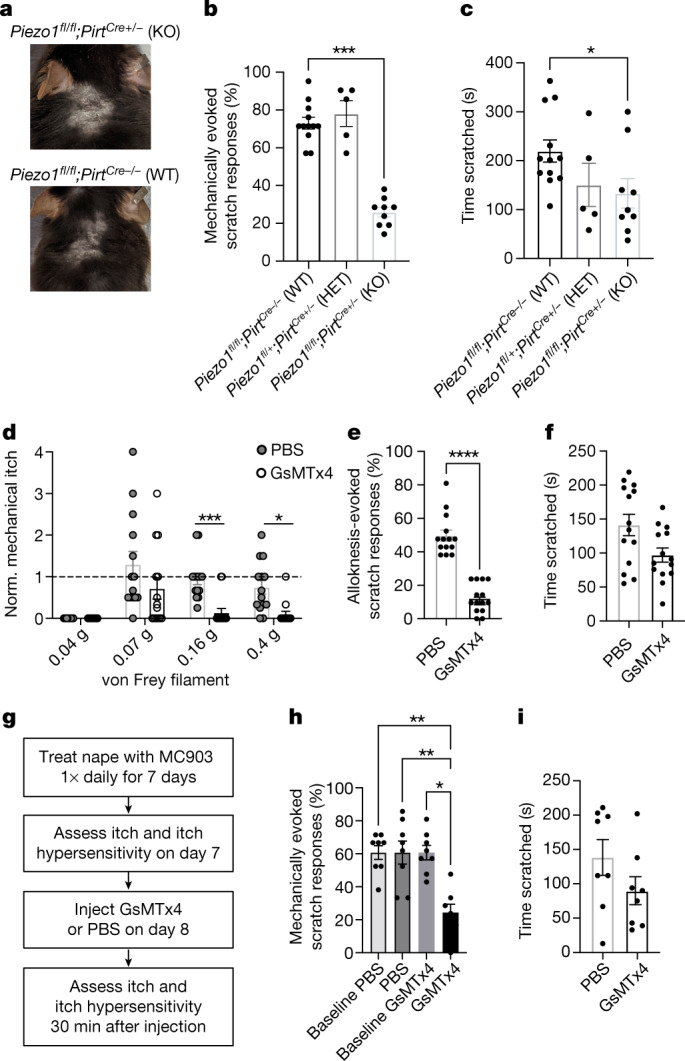
Neuronal PIEZO1 mediates itch hypersensitivity in a mouse model of chronic itch. **a**, Representative images (*n* = 12 WT and *n* = 9 KO mice from two experiments) of nape skin of *Piezo1 * ^*fl/fl*^;*Pirt*^*Cre+/−*^ (KO; top) and *Piezo1* ^*fl/fl*^;*Pirt*^*Cre*−/−^ (WT; bottom) littermates on day 8 of the MC903 model. **b**, MC903-evoked mechanical itch hypersensitivity in *Piezo1*^*fl/fl*^ or *Piezo1*^*fl/+*^*;Pirt*^*Cre−/−*^ (WT), *Piezo1*^*fl/+*^*;Pirt*^*Cre+/−*^ (HET) and *Piezo1*^ *fl/fl*^*;Pirt*^*Cre+/−*^ (KO) mice (Kruskal–Wallis: ***P = 0.0002, *χ*^2^ = 17.36; Dunn’s: ***P_adjusted_ = 0.0004). **c**, MC903 spontaneous scratching (Kruskal–Wallis: *P = 0.0444, *χ*^2^ = 6.231; Dunn’s: *P_adjusted_ = 0.0319). Data in **b**,**c** are from *n* = 12 WT, *n* = 5 HET and *n* = 9 KO mice from two experiments. **d**, Mechanically evoked scratching after injection of phosphate-buffered saline (PBS) or GsMTx4 in wild-type mice, normalized to baseline; see also Extended Data Fig. 9b (three-way ANOVA: ****P_treatment_ < 0.0001, *F*(1, 104) = 51.38; Tukey’s *P*_adjusted_: ***P_0__.16g_ = 0.0002, *P_0.4g_ = 0.0260; *n* = 14 mice). **e**, Histamine alloknesis (Mann–Whitney: ****P < 0.0001, *U* = 0; *n* = 14 mice). **f**, Histamine-evoked scratching (Mann–Whitney: *P* = 0.0709, *U* = 58.50; *n* = 14 mice). Data in **d**–**f** are from two experiments. **g**, Schematic of MC903 chronic itch model experiments with acute GsMTx4. **h**, MC903 itch hypersensitivity before and after injection of PBS or GsMTx4 (Kruskal–Wallis: **P = 0.0013, *χ*^2^ = 15.66; Dunn’s (left to right): **P_a__djusted_ = 0.0063, **P_adjusted_ = 0.0066, *P_adjusted_ = 0.01; *n* = 8 mice). **i**, MC903 spontaneous scratching after injection of PBS or GsMTx4 (Mann–Whitney: *P* = 0.1848, *U* = 19; *n* = 8 mice). Data in **h**–**i** are from two experiments. Error bars represent mean ± s.e.m. of *n* biological replicates (mice) and statistical tests are two-tailed where applicable. Source data

**Extended Data Fig. 9:**
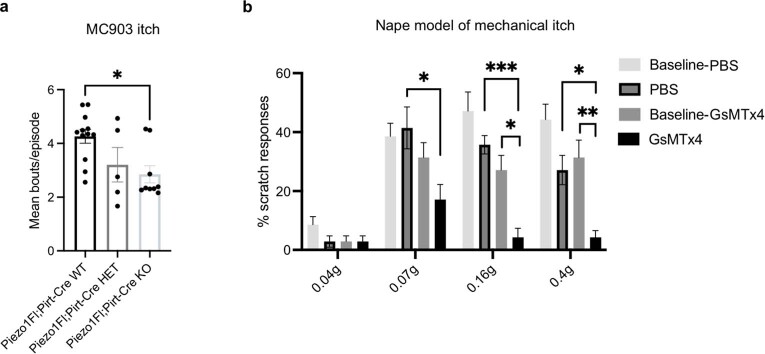
Additional MC903 and GsMTx4 scratching behavioural data. **a**, Mean bouts per scratch episode from *Piezo1*^*fl/fl*^*; Pirt*^*Cre*^ mice shown in Fig. 4c (Kruskal–Wallis: *p = 0.0359; χ^2^ = 6.656; Dunn’s *p_adjusted_ = 0.0279). Data are from *n* = 12 WT, *n* = 5 HET, and *n* = 9 KO mice from 2 experiments. **b**, Non-normalized data from the nape mechanical itch assay replotted with values and statistical test results (* symbols) representing *P* values from Fig. 4d (*n* = 14 mice from 2 experiments). Error bars represent mean ± s.e.m. of *n* biological replicates (mice) and statistical tests are two-tailed where applicable. Source data

We wondered whether PIEZO1-dependent mechanical itch is relevant to chronic itch, a global health issue with a lifetime prevalence exceeding 10% in humans^40^. We tested the relevance of PIEZO1-dependent mechanical itch in a widely used mouse model of chronic itch that has been shown to mimic specific aspects of human atopic dermatitis, the most common chronic itch disorder^41^. Daily application of the vitamin D analogue MC903 (calcipotriol) induces profound erythema, xerosis, excoriation, itch-evoked scratching and itch hypersensitivity in the mouse nape, ear or cheek^42^. We observed the development of mature lesions in wild-type and *Piezo1*^*fl/fl*^*;Pirt*^*Cre+/−*^ mice that were treated with MC903, which suggests that skin inflammation develops normally in the absence of neuronal PIEZO1 (Fig. 4a). Of note, knockout mice showed significant deficits in mechanical itch hypersensitivity (Fig. 4b), with mildly but significantly decreased spontaneous scratching behaviours compared to control littermates treated with MC903 (Fig. 4c), which were largely explained by a reduced number of scratch bouts per episode (Extended Data Fig. 9a). This suggests that partially independent mechanisms underlie itch hypersensitivity versus spontaneous scratching in the setting of chronic itch, and aligns with previous work showing that diverse endogenous chemical pruritogens that are released from immune and skin cells contribute to itch in this model^42^.

Finally, we asked whether acute inhibition of PIEZO1 could alleviate mechanical itch and itch sensitization. We investigated whether inhibition of PIEZO1 with the toxin GsMTx4 could phenocopy the genetic loss of PIEZO1 (refs. ^39,43^). Indeed, pretreatment with intraperitoneal injection of GsMTx4 to achieve systemic blockade^39^ reduced mechanically evoked scratching in naive mice (Fig. 4d and Extended Data Fig. 9b) and alloknesis after histamine injection (Fig. 4e), but did not significantly affect histamine-evoked itch (Fig. 4f). Furthermore, MC903-dependent itch hypersensitivity in the nape was largely attenuated in GsMTx4-treated mice (Fig. 4g,h), whereas spontaneous scratching behaviours persisted, albeit at slightly reduced levels (Fig. 4i). Although GsMTx4 is not a selective PIEZO1 antagonist and inhibits other mechanically activated channels in vitro^44^, when taken together with our chronic itch data from PIEZO1 conditional KO mice, this finding indicates that PIEZO1 antagonists have a potential use in the treatment of itch.

## Discussion

**Extended Data Fig. 10:**
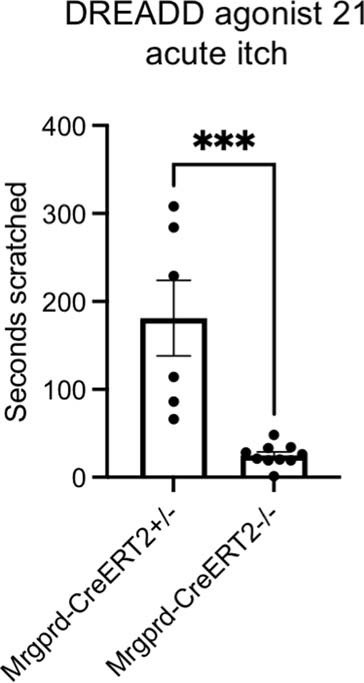
Chemogenetic activation of MRGPRD^+^ neurons evokes scratching. Cheek model of DREADD agonist 21-evoked itch in *HM3dGq*^ *fl/fl*^*; Mrgprd*^*CreERT2+/*-^ and ^-/-^ mice (Mann–Whitney: ***p = 0.0002; U = 0, *n* = 6,10 mice from 1 experiment). Error bars represent mean ± s.e.m. of *n* biological replicates (mice) and statistical tests are two-tailed where applicable. Source data

In summary, our work shows that a subset of itch-sensing neurons are polymodal, responding to chemical and mechanical pruritogens. The PIEZO1^+^ pruriceptors described here may act either in parallel with or independently of previously identified LTMR-dependent mechanical itch circuitry^2^. The question remains as to why PIEZO1 transduces mechanical itch when PIEZO2 is expressed in somatosensory neurons and is exquisitely sensitive to mechanical stimuli. We speculate that PIEZO1-dependent mechanical itch in slow-conducting C fibres may fuel the persistent sensation of a burrowing parasite and drive the desire to scratch until the organism is expelled from the skin, whereas Aβ-LTMR-dependent mechanical itch may have a key role in coordinating and triggering a rapid reflexive response, much like how PIEZO2^+^ LTMRs coordinate nociceptive reflexive behaviours (such as response to pinprick) that are largely independent of PIEZO2 (ref. ^45^). Moreover, previous in vitro work has shown that PIEZO1 is more sensitive to membrane stretch (through suction stimulation of the membrane patch in cell-attached mode) than PIEZO2 (ref. ^46^). Although we did not test the membrane stretch responsiveness of SST^+^ neurons, one hypothesis is that the distinct mechanical activation properties of PIEZO1 may favour the mechanosensitivity of free nerve endings like those of pruriceptors, whereas the properties of PIEZO2 may specifically favour specialized touch-sensitive end organs. In addition, although we show that PIEZO1 has an essential role in SST^+^ neurons in mechanical itch, we cannot rule out the possibility that MRGPRD^+^ neurons contribute to mechanical itch—despite their controversial role as itch mediators^47^. With regard to this point, we did observe itch-evoked scratching in mice with chemogenetic activation of mature MRGPRD^+^ neurons expressing the hM3Dq DREADD (designer receptor exclusively activated by a designer drug) after the injection of DREADD agonist 21 into the cheek (Extended Data Fig. 10), supporting previous studies that implicated MRGPRD^+^ neurons in itch^21^. The contribution of PIEZO2 to mechanical itch remains unclear given the opposing effects of different LTMR subpopulations on mechanical itch (that is, Merkel cell afferents versus TLR5^+^ LTMRs)^14,17^. Highly selective genetic strategies will need to be developed to investigate how PIEZO1- and PIEZO2-dependent itch pathways intersect, as the present methods of accomplishing the knockout of PIEZO2 in LTMRs also target proprioceptors that coordinate reflexive behaviours^37^, which may confound the study of itch-evoked scratching.

On a final note, the genetics of itch are largely attributed to variants in a handful of genes that are mainly found in white European populations, which do not contribute substantially to itch disorders in Black or African populations^48^. Given the significance of *PIEZO1* variants to human health, particularly in underserved and understudied Black and African populations^4,39^, it will be important to examine whether and how variation in *PIEZO1* contributes to itch. Such studies will require large-scale genomics databases complemented with extensive clinical phenotyping of mechanical itch and chronic itch severity, which has so far been performed only on small cohorts of individuals owing to the complexity of such experiments.

## Methods

### Statistics

All statistical analyses were performed in Prism 9.3.0 (GraphPad). Error bars are defined as the mean ± s.e.m unless otherwise indicated, and wherever feasible, individual data points or total counts are plotted. For Fig. 3b,e,k, and Extended Data Figs. 7k,l, 8i and 9b, only mean ± s.e.m is plotted owing to the large number of columns, and individual values are provided in the Source Data. All tested covariates are reported in the legends. Two-tailed tests were performed wherever applicable. *n* numbers, test statistics, exact *P* values and degrees of freedom are indicated in the figure legends. Aside from electrophysiology data, which were analysed using previously described methods^3,18^, normality and/or equal variance were not assumed and so nonparametric tests were used throughout, with the appropriate post-hoc test indicated for multiple comparisons. When two or more independent variables were examined, a two- or three-way analysis of variance (ANOVA) was used, and sphericity was not assumed.

### Study design

No analyses were performed in advance to pre-determine sample size. Sample sizes were based on similar studies in the literature^14,18^. All attempts at replication were successful. All experiments were repeated more than once as indicated in the figure legends except for Extended Data Figs. 7r, 8a and 10, and *n* numbers (biological replicates) are indicated for those experiments in the figure legends. For those experiments that were repeated only once, it is stated as such in the figure legend. No randomization was used. Mice were arbitrarily assigned to treatment and vehicle groups for the GsMTx4 and Yoda1 experiments, as they were of identical age, genotype and sex, so no randomization was possible. For all other behaviour experiments, entire cohorts or litters of mice were tested at once by a blinded experimenter, so no allocation or randomization was needed or possible. Mice were arbitrarily assigned behavioural chamber numbers by the blinded experimenter. For all behavioural experiments, the experimenter and scorer (analyser) was blinded whenever possible to both treatment (when two or more treatments were applied) and/or genotype (when two or more genotypes were tested). For electrophysiology and calcium imaging, a single coverslip or chamber of cells from each genotype or condition was tested in alternating order with the opposing genotype or condition (for example, siRNA or drug treatment) so that genotypes and conditions were assessed in parallel. For all other experiments, no randomization was needed or possible as there were no conditions to compare between. For calcium imaging, data were analysed offline using automated routines and so blinding was not necessary. For electrophysiology, experiments were conducted as previously published without blinding^3,18,37^. For all other experiments, there were no comparisons so blinding was unnecessary.

### Mice

All experiments were performed under the policies and recommendations of the International Association for the Study of Pain and approved by the Scripps Research Animal Care and Use Committee. Mice were kept in standard housing with a 12-h light–dark cycle set with lights on from 6 am to 6 pm, with the room temperature kept around 22 °C, and humidity between 30% and 80% (not controlled). Mice were kept on pelleted paper bedding and provided with paper square nestlets and polyvinyl chloride pipe enrichment with ad libitum access to food and water. Age-matched littermate mice were used for all in vivo experiments. For all in vivo experiments except for Figs. 3h,i and 4d–i, which used only male mice, male and female mice were used and pooled. Mouse ages ranged from 2 to 6 months for behavioural studies, and 1.5 to 4 months for electrophysiology, calcium imaging, IHC and smFISH. The homozygous *Piezo1*^*tdTomato*^ mice were previously described^25^ and were maintained in the laboratory (*B6;129-Piezo1*^*tm1.1Apat/J*^; Jackson Laboratories 029214). The *HM3dGq* ^*fl/fl*^*;Mrgprd* ^*CreERT2+/*−^ mice were generated by crossing commercially available *HM3dGq* ^*fl/fl*^ mice (B6N;129-Tg(CAG-CHRM3*,-mCitrine)1Ute/J; Jackson Laboratories 026220) with *Mrgprd*^ *CreERT2+/−*^ mice (*Mrgprd *^*tm1.1(cre/ERT2)Wql*^; Jackson Laboratories 031286), and intercrossing the progeny to obtain the desired genotypes. Recombination was achieved with once-daily intraperitoneal injection of 75 mg per kg body weight tamoxifen (Sigma) dissolved in 0.22-µm sterile-filtered corn oil delivered to both experimental and control mice over five consecutive days. The *Ai9* ^*fl/fl*^*;Sst*^*Cre+/−*^ mice were generated by crossing commercially available *Ai9* ^*fl/fl*^ female mice (*B6.Cg-Gt(ROSA)26Sor*^*tm9(CAG-tdTomato)Hze/J*^; Jackson Laboratories 007909) with *Ai9*^*fl/fl*^*; Sst*^*Cre+/+*^ males (*B6J.Cg-Sst*^* tm2.1(cre)Zjh/MwarJ*^; Jackson Laboratories 028864). Visibly pink or red mice were not used for experiments, as some germline recombination was observed. *Piezo1* ^* fl/fl*^;*Sst*^* Cre+/−*^ mice were also generated from this line. *Piezo1 * ^*fl/fl*^*;Pirt*^*Cre+/*^ mice were generated by crossing *Piezo1* ^*fl/fl*^ female mice (*Piezo1 * ^*tm2.1Apat/J*^; Jackson Laboratories 029213) with *Pirt*^*Cre+/−*^ males (*Pirt*^*tm3.1(cre)Xzd*^, gift from X. Dong, Johns Hopkins University), and then crossing the *Piezo1* ^* fl/+*^*;Pirt*^* Cre+/−*^ male offspring with *Piezo1 *^*fl/fl*^ or *Piezo1*^* fl/+*^*;Ai9*^ *fl/+*^ or *Ai9 *^*+/+*^ female mice to generate homozygous knockouts, heterozygous mice and *Pirt*^*Cre−/−*^ control mice, some of which carried the *Ai9 *^ *fl/+*^ allele. The PIEZO1^GOF^ mouse line ubiquitously carries the nucleotide change c.GG_7742-7743_AC and has been previously described^4^. Experimental PIEZO1^GOF^ mice were generated from heterozygous matings. These above strains were maintained on a C57BL6/J background when not intercrossed to generate desired genotypes, except for *Piezo1*^*tdTomato*^ and *Mrgprd*^*CreERT2+/−*^, which were maintained as inbred stocks. C57BL6/J wild-type male mice used in Figs. 3h,i and 4d–i were purchased from the Scripps Research Department of Animal Resources rodent breeding colony. PCR genotyping from tail snip DNA samples was performed in-house using guidelines from Jackson Laboratory. All mice except for purchased C57BL6/J mice received metal identification tags (National Band & Tag, 1005-1) on the right ear when they were between 18 and 30 days old. After weaning between 21 and 30 days of age, mice were co-housed in groups of 2–5 littermates of the same sex.

### smFISH

For mouse experiments, DRG tissues were removed immediately, embedded in optimal cutting temperature compound (OCT, Sakura), and flash-frozen in liquid nitrogen. For human tissue, a flash-frozen T1 (thoracic)-level DRG was obtained from Anabios from one female donor aged 45 with no history of neurological disease. The human DRG was embedded into pre-chilled OCT over dry ice such that it remained frozen, and 20-µm cryosections were used for all experiments. The protocol for RNAscope Multiplex Fluorescent Reagent Kit V2 (ACDBio: 323100) was followed exactly according to the instructions for fresh-frozen tissue. Protease IV was applied for 22 min for mouse tissue and 30 min for human tissue after pilot experiments to optimize protease conditions. Probes (all from ACDBio) for mouse *Piezo1* (C1; 400181), mouse *Piezo2* (C1; 400191, C2; 400191-C2), mouse *Mrgprd* (C3; 417921-C3), mouse *Nppb* (C3; 425021-C3), mouse *Mrgpra3* (C2; 548161-C2), mouse *Calca* (C2; 417961-C2), mouse *Scn10a* (C2; 426011-C2), human *PIEZO1* (C1; 485101), human *PIEZO2* (C1 or C2; 449951, 449951-C2), human *NPPB* (C2; 448511-C2) and *tdTomato* (C2; 317041-C2) were applied to detect transcript. Quantification of images was performed manually in ImageJ (Fiji, 2.3.0/1.53f) using regions of interest (ROIs) to define the quantification area. In mouse tissues, positive ROIs were counted as those with more than five puncta per ROI, on the basis of experiments with the 3-plex *Dapb* negative control probe (320871). Cell borders were drawn around highly expressed marker transcript signals to define individual cells. In humans, cells with more than three puncta per ROI were counted as positive cells, based on the *Dapb* negative control probe. Cell borders were drawn around highly expressed marker transcript signals to define individual cells, and cells needed to have a clearly defined satellite glial border. Lipofuscin was present in human DRGs, and those areas were identified by identical fluorescence signals across multiple detection channels using published criteria^49^, and so puncta were not counted in those regions. Displayed images were uniformly cropped from the original 20× images on which quantification was performed.

### Immunohistochemistry

For PIEZO1^tdTomato^ experiments, tissues were processed using a modified protocol to preserve signal^50^. In brief, fresh-frozen DRGs and trigeminal ganglia were embedded in OCT and sectioned at 20 µm. Sections were post-fixed on slides in cold 4% paraformaldehyde (PFA) in PBS for 10 min at room temperature and quenched using 20 mM glycine and 75 mM ammonium chloride with 0.1% v/v Triton X-100 in PBS for 10 min. Slides were washed in PBS and then incubated in blocking buffer (0.6% w/v fish skin gelatin with 0.05% w/v saponin in PBS with 5% v/v normal goat or donkey serum) for 1 h at room temperature. Slides were incubated in primary antibodies overnight at 4 °C in blocking buffer without serum: 1:200 rabbit anti-RFP (Rockland 600-401-379), 1:200 rat anti-PECAM1 (Sigma CBL1337-I) and 1:1,000 chicken anti-NefH (Abcam ab4680). Slides were washed in PBS, and then incubated in secondary antibodies in blocking buffer 1 h at room temperature (all 1:1,000): goat anti-rabbit AlexaFluor 594 (Life Technologies A11037), donkey anti-rat AlexaFluor 488 (Jackson 712-546-153) and donkey anti-chicken AlexaFluor 647 (Jackson 703-605-155). Samples were mounted in SlowFade Diamond and sealed with nail polish. For conventional IHC, mice were transcardially perfused with 15–30 ml ice-cold PBS followed by 30 ml 4% PFA in PBS. DRGs were dissected into PBS and post-fixed for 20 min on ice in 4% PFA in PBS. Tissues were cryoprotected overnight in 30% sucrose-PBS (w/v) at 4 °C before embedding in OCT and sectioning at 20 µm on a cryostat. Sections were briefly rinsed in PBS, washed for 10 min in 0.3% Triton X-100 in PBS (PBST), then blocked for 1 h in 5% normal goat serum in 0.3% PBST. Sections were incubated for 2 h at room temperature in rabbit anti-CGRP (Immunostar 24112) diluted 1:1,000 in 0.3% PBST. Sections were washed in PBS and incubated in 1:1,000 goat anti-rabbit AlexaFluor 488 (Thermo Fisher Scientific A32731) and 25 µg ml^−1^ isolectin B4 AlexaFluor 647 conjugate (Life Technologies I32450) for 1 h at room temperature. Tissues were rinsed in PBS, mounted in Fluoromount G + DAPI (4’,6-diamidino-2-phenylindole) and sealed with nail polish. For both smFISH and IHC, all samples were imaged on either a Nikon A1 or a Nikon C2 confocal microscope and the imaging settings (laser power, gain, 1,024 × 1,024 original resolution, pixel dwell, objective and use of Nyquist zoom) were kept consistent within experiments. For all images, brightness and contrast adjustments were uniformly applied to the entire image.

### Cell culture

Cell culture was carried out as previously described^18^. In brief, DRGs were dissected and incubated for 60 min at 37 °C in 6.25 mg ml^−1^ collagenase IV (Life Technologies 17104-019) in serum-free medium, followed by incubation in 1 U ml^−1^ papain (Fisher NC9199962) for 30 min at 37 °C. Cells were triturated and transferred into medium with 10% fetal bovine serum supplemented with the following growth factors (from Gibco): GDNF 50 ng ml^−1^, NGF 100 ng ml^−1^, NT-4 50 ng ml^−1^, NT-3 50 ng ml^−1^ and BDNF 50 ng ml^−1^. For calcium imaging, 10 µM cytosine arabinoside (Sigma) was added to the medium. Cells were plated onto poly-d-Lysine and laminin-coated glass coverslips (Corning, for electrophysiology) or eight-well chambered coverslips (Ibidi, for calcium imaging). Media: HyClone DMEM/F12 1:1 with l-glutamine and HEPES (Cytiva or Gibco) supplemented with 1:100 penicillin–streptomycin (Gibco). For calcium imaging, cells were used within one to three days. For nucleofection experiments, cells were used three to five days after plating. DRG cultures and transfection of siRNA were performed exactly as described^3,37^ using the Amaxa P3 Primary Cell 4D-Nucleofector X Kit S (Lonza, V4XP-3032); 120 pmol of siRNA and 400 ng of pIRES2-eGFP (Clontech 6029-1) plasmid were nucleofected per reaction. Reagents: mouse *Piezo1* siRNA (ON-TARGETplus mouse *Piezo1* (234839) siRNA SMARTpool; L-061455-00-0005), mouse *Piezo2* siRNA (ON-TARGETplus mouse *Piezo2* (667742) siRNA SMARTpool; L-163012-00-0005) and non-targeting siRNA (ON-TARGETplus non-targeting siRNA; D-001810-10-05).

### Calcium imaging

Cells were loaded for 60 min at room temperature with 10 µM Fura-2AM (Life Technologies F1201) supplemented with 0.01% Pluronic F-127 (w/v; Life Technologies) in a physiological Ringer’s solution containing 127 mM NaCl, 3 mM KCl, 10 mM HEPES, 2.5 mM CaCl_2_, 1 mM MgCl_2_ and 10 mM D-(+)-glucose, pH 7.3. All chemicals were purchased from Sigma. Neurons were presented with 20 µM Yoda1 (Tocris) in 1% DMSO-Ringer’s vehicle, 100 µM histamine dihydrochloride (Tocris) in Ringer’s, 1 mM β-alanine (Tocris) in Ringer’s and/or 100 µM allyl isothiocyanate (AITC, Sigma) in 0.1% DMSO-Ringer’s. Images were acquired using MetaFluor software (v.7.8.2.0) and displayed as the ratio of 340 nm/380 nm. Cells were identified as neurons by eliciting depolarization with high-potassium Ringer’s solution (71.5 mM) at the end of each experiment. Responding neurons were defined as those having an increase of more than 15% from the baseline ratio. Analysis was performed using previously established methods in Igor Pro 6.3.7 (WaveMetrics)^51,52^. Fifty-seven individual neurons with compound addition artefacts (large spikes in the calcium imaging trace) were excluded from the area under the curve analysis but were still used for the peak normalized ratio analysis. In separate experiments, cells were incubated for 5 min in a sub-threshold 1 µM histamine (which did not elicit calcium transients), before stimulation with 20 µM Yoda1. Fura2 ratios were normalized to the baseline ratio *F*_340_/*F*_380_ = (Ratio)/(Ratio_*t*=0_).

### Electrophysiology

#### siRNA knockdown of Piezo genes

Whole-cell patch clamp recordings were performed using an Axopatch 200B amplifier as described using standard methods to achieve an access resistance of 6.6 ± 0.2 MΩ (*n* = 186)^3,18^. During recording, cells were maintained at 21–23 °C in physiological Ringer’s solution and clamped at −80 mV. Electrodes had resistances of 3.4 ± 0.1 MΩ (*n* = 186) when filled with gluconate-based low-chloride intracellular solution: 100 mM K-gluconate, 25 mM KCl, 0.483 mM CaCl_2_, 3 mM MgCl_2_, 10 mM HEPES, 1 mM BAPTA tetrapotassium salt, 4 mM Mg-ATP and 0.4 mM Na-GTP (pH 7.3 with KOH). Neuronal somata were tested for mechanosensitivity using a fire-polished glass probe. The probe displacement was advanced in increments of 1 μm using a computer-controlled piezoelectric stimulator^3,18^. All data were analysed as previously described^3,18,37^ using pClamp 10 and Prism 9.3.0.

#### Histamine sensitization of mechanically activated currents

Whole-cell patch clamp recordings were performed in parallel by two experimenters using either an Axopatch 200B amplifier or a Multiclamp 700A amplifier. Baseline mechanically activated currents were measured as described above using increasing 0.5-µm displacement increments until the stimulus-intensity–response relationship approached *I*_max_. Histamine dihydrochloride (100 µM; Tocris) was delivered by gravity perfusion at a rate of 2–3 ml min^−1^. Mechanically activated currents were assessed again in the presence of histamine, which elicited an inward current. Washout of histamine was performed over several minutes. Cells were finally perfused with 10 µM Yoda1 to assess whether the inactivation kinetics of the mechanically activated currents were slowed as has been previously shown for heterologous PIEZO1 currents^26^.

### Behavioural studies

All behavioural experiments were performed between 12:00 and 18:00 in the same room. When multiple tests were performed on a cohort of mice (as in Fig. 3), tests were performed in the following order over the course of seven days: von Frey series, up–down von Frey, pinprick, Randall–Selitto, tail clip, mechanical itch and acute histamine itch followed by measurement of alloknesis. With the exception of the MC903 model, in which cagemates could ingest the topically applied chemical and so were singly housed, mice were co-housed with one to four littermates of the same sex. Mice with noticeable lesions, wounds, ear chondritis or poor physical condition were not used for behavioural studies. The experimenter and scorer(s) (for itch) were blind to the genotype and the compound injected (where relevant).

#### Itch-evoked scratching behaviour

Itch and acute pain behavioural measurements were performed as previously described^51,53,54^. Mice were shaved on the nape of the neck, the fluffy hairs of the back of the left ear, or the right cheek five to seven days before the experiment with surgical clippers under 1–2% isoflurane. Unless indicated otherwise, all itch behaviours were performed in the nape. For all itch behaviour experiments, mice were acclimated in the behaviour chambers on the two days before behavioural measurements for one hour. For chemical itch experiments, compounds (histamine dihydrochloride: 50 µg in PBS, Tocris; Yoda1: 14.2 ng, 142 ng or 355 ng in 1% DMSO-PBS, Tocris; chloroquine diphosphate: 200 µg in PBS, Tocris; recombinant mouse IL-31: 60 pmol in 0.9% NaCl, Peprotech; DREADD agonist 21: 25 µg in PBS, Tocris) were injected via the intradermal route using a 31g insulin syringe in a total volume of 20 µl. Mice were individually placed into covered four-part plexiglass chambers with opaque dividers (Ugo Basile) on a plexiglass platform (Fab Glass and Mirror) with a small square of paper bedding to absorb excess urine. Bout and episode quantity, and episode length, were manually scored from videos recorded with either a GoPro Hero 8 camera or a Nikon D3200 camera. All behaviour videos were recorded from below using a mirror and scored for 30 min, except for Fig. 4f,i that were recorded and scored for 25 min. Behavioural scoring was performed using QuickTime 10.4. A scratch episode was defined as a period of one or more scratching bouts from the moment the paw was lifted from the plexiglass floor to when it was returned, or paw grooming persisting for three or more seconds. A bout was defined as a series of one or more scratches within an episode in which the paw was lifted towards and then away from the site of scratching. Wipes were defined as unilateral forepaw motions on the cheek that did not occur during a period of facial grooming (in which the face is wiped with both paws).

#### Mechanical itch

Mechanical itch experiments were performed similar to previously described methods^14,32^. Mice were placed into four-part plexiglass chambers on a plexiglass platform and acclimated for 30 min. From above, the mice were probed on the shaved nape or the shaved back of the left ear (Extended Data Fig. 7k) with a descending force-series of five trials per force using von Frey monofilaments (Touch Test) ranging from 1 g to 0.008 g. A positive response was scored as one or more instances of site-directed scratching with the hind paw. Mice that were spontaneously scratching were not probed until 1 min after scratch cessation. Cumulative scratch responses report the total number of scratch responses divided by the total number of trials as a percentage, regardless of filament force.

#### Alloknesis models

In the histamine- and Yoda1-evoked alloknesis models, itch-evoked scratching was recorded immediately after injection of 50 µg histamine, 60 pmol IL-31 or 355 ng Yoda1 for 30 min before assessment. The shaved nape was probed for mechanical itch responses (see above) with the 0.04 g filament three times in a 5-min interval for a total of 30 min (21 tests in total)^14,32^.

#### MC903-induced chronic itch

The MC903 model of chronic itch was performed as previously described^42,51^. In brief, mice were shaved on the nape and singly housed five days before the start of the model. MC903 (0.2 mM; Calcipotriol, Tocris) was prepared fresh in absolute ethanol, and 20 µl was applied using a micropipette to the skin each morning between 07:00 and 09:00. On day 8, spontaneous scratching was recorded for 30 min before the assessment of itch hypersensitivity using identical methods to the above alloknesis method.

#### GsMTx4 experiments

GsMTx4 was acquired from Abcam, prepared fresh in sterile PBS and injected intraperitoneally at 540 µg per kg body weight^39^. Baseline mechanical itch was assessed the day before injection using an attenuated filament series from 0.4 g to 0.04 g. The following day, GsMTx4 or PBS vehicle was injected, and histamine-evoked scratching was recorded for 25 min 1 h after injection. Histamine alloknesis was assessed immediately afterwards. For MC903-induced itch behaviours, baseline mechanical itch hypersensitivity was assessed on day 7 of the model. On day 8, GsMTx4 was administered 1 h before the itch-evoked scratching measurements (recorded for 25 min) and the itch hypersensitivity assay described above.

#### von Frey assays

The mechanical threshold was measured using calibrated von Frey monofilaments (Touch Test) on a metal mesh platform (Ugo Basile). von Frey experiments were performed as previously described using the up–down method starting with 1 g, or a descending force-series of four trials per force from 4 g to 0.008 g (refs. ^18,37^). Valid responses included fast paw withdrawal; licking, biting or shaking of the affected paw; or flinching. Mice were allowed to acclimate on the platform for 1 h before measurements. For von Frey mechanical allodynia behaviour, 355 ng Yoda1 was injected into the plantar surface of the hind paw and the mechanical threshold was quantified using the up–down method just before injection, and 5 min, 15 min and 30 min after injection.

#### Pinprick

The pinprick assay was conducted on the von Frey testing platform. The mouse hind paw was poked with a 27 g syringe needle without breaking the skin to induce fast acute mechanical pain^18,37^. Each paw was stimulated 10 times with the needle, with a 5-min rest in between trials, and the per cent withdrawal (fast withdrawal; licking, biting or shaking of paw; jumping; and/or flinching) was calculated from the total number of trials. For latency measurements, the assay was performed just as above, except that the needle was soldered to a braided copper wire that was connected by a BNC cable to a standard digital oscilloscope (Tektronix). Using the 'trigger' mode, the duration of the voltage trace was used to determine how long the paw was in contact with the filament to determine the latency to withdrawal.

#### Tail clip

The tail clip assay was performed as previously described^18,37^. Mice were acclimated on a metal benchtop for 15 min in clear circular plexiglass chambers before assessment. The alligator clip was placed near the base of the mouse tail. A response was scored when the mice showed awareness of the clip by biting, vocalization, grasping of tail or a jumping response. Latency was measured with a stopwatch, with a minimum recordable time of 1 s.

#### Randall–Selitto

The Randall–Selitto assay was performed as previously described^18^. In brief, mice were gently restrained in the hand of the experimenter and a pinching force was applied to the hind paw using a Randall–Selitto device (IITC Life Sciences). A 300-g cut-off was used. A response was scored by any visible flinching of the hind limb or audible vocalization.

#### Proprioception assay

In brief, naive adult mice were restrained by the tail and held over the countertop or home cage and the hind limbs were photographed. A 0–2 scoring system was developed, in which images of a *Piezo2* ^*fl/fl*^*;Hoxb8*^ *Cre+/−*^ mouse^37^ represented '0', or severe proprioceptive deficit; images of a C57BL6/J mouse represented '2', or normal; and any intermediate or uncertain images were scored a '1', which could have been indicative of transient limb positioning from a proprioceptively normal mouse or a mild compromise in proprioception. Images were scored by five independent, blinded scorers and the results of each experimenter were averaged for each mouse.

### Histology

Yoda1 (355 ng) or vehicle was injected intradermally into the shaved nape skin of C57BL6/J mice. Mice were euthanized 30 min after injection, the skin was de-haired with depilatory cream (Nair) and then rinsed with water, and the section of back skin immediately around the site of injection was dissected and fixed in 10% formalin for paraffin embedding, sectioning and haematoxylin and eosin (H&E) staining. Skin sections were imaged at 20× using a Keyence microscope.

### Reporting summary

Further information on research design is available in the Nature Research Reporting Summary linked to this paper.

## Online content

Any methods, additional references, Nature Research reporting summaries, source data, extended data, supplementary information, acknowledgements, peer review information; details of author contributions and competing interests; and statements of data and code availability are available at 10.1038/s41586-022-04860-5.

## Supplementary information

Reporting Summary

## Data Availability

Raw data are available from the authors upon reasonable request. The previously published single-cell RNA-seq data shown in Extended Data Fig. 1 are available at: https://kleintools.hms.harvard.edu/tools/springViewer_1_6_dev.html?datasets/Sharma2019/all. Source data are provided with this paper.

## Source data

Source Data Fig. 2
Source Data Fig. 3
Source Data Fig. 4
Source Data Extended Data Fig. 6
Source Data Extended Data Fig. 7
Source Data Extended Data Fig. 8
Source Data Extended Data Fig. 9
Source Data Extended Data Fig. 10
