# Supplementary material for: PIEZO1 transduces mechanical itch in mice
Source: Nature. 2022 Jun 22;607(7917):104–10. doi: 10.1038/s41586-022-04860-5 (PMC9259491; doi:10.1038/s41586-022-04860-5)
Supplement: Supplementary file 1 — Reporting Summary [file 41586_2022_4860_MOESM1_ESM.pdf]

nature portfolio

Corresponding author(s): Ardem Patapoutian

Last updated by author(s): May 1, 2022

Reporting Summary

Nature Portfolio wishes to improve the reproducibility of the work that we publish. This form provides structure for consistency and transparency in reporting. For further information on Nature Portfolio policies, see our Editorial Policies and the Editorial Policy Checklist.

Statistics

For all statistical analyses, confirm that the following items are present in the figure legend, table legend, main text, or Methods section.

n/a	Confirmed	
☐	☒	The exact sample size (*n*) for each experimental group/condition, given as a discrete number and unit of measurement
☐	☒	A statement on whether measurements were taken from distinct samples or whether the same sample was measured repeatedly
☐	☒	The statistical test(s) used AND whether they are one- or two-sided<br>*Only common tests should be described solely by name; describe more complex techniques in the Methods section.*
☐	☒	A description of all covariates tested
☐	☒	A description of any assumptions or corrections, such as tests of normality and adjustment for multiple comparisons
☐	☒	A full description of the statistical parameters including central tendency (e.g. means) or other basic estimates (e.g. regression coefficient) AND variation (e.g. standard deviation) or associated estimates of uncertainty (e.g. confidence intervals)
☐	☒	For null hypothesis testing, the test statistic (e.g. *F*, *t*, *r*) with confidence intervals, effect sizes, degrees of freedom and *P* value noted<br>*Give P values as exact values whenever suitable.*
☒	☐	For Bayesian analysis, information on the choice of priors and Markov chain Monte Carlo settings
☒	☐	For hierarchical and complex designs, identification of the appropriate level for tests and full reporting of outcomes
☒	☐	Estimates of effect sizes (e.g. Cohen's *d*, Pearson's *r*), indicating how they were calculated

Our web collection on statistics for biologists contains articles on many of the points above.

Software and code

Policy information about availability of computer code

Data collection	Electrophysiology data were collected using pClamp 10 (Molecular Devices). Calcium imaging data were collected using MetaFluor 7.8.2.0 (Molecular Devices).
Data analysis	Statistical tests were performed in Prism 9.3.0 (GraphPad). Calcium imaging data were analyzed in IgorPro 6.3.7 (WaveMetrics). Electrophysiology data were analyzed in pClamp 10 (Molecular Devices). Behavioral videos were scored using QuickTime 10.4. smFISH images were analyzed in ImageJ (FIJI, 2.3.0/1.53f). smFISH and IHC images were prepared for publication using ImageJ (FIJI, 2.3.0/1.53f).

For manuscripts utilizing custom algorithms or software that are central to the research but not yet described in published literature, software must be made available to editors and reviewers. We strongly encourage code deposition in a community repository (e.g. GitHub). See the Nature Portfolio guidelines for submitting code & software for further information.

Data

Policy information about availability of data

All manuscripts must include a data availability statement. This statement should provide the following information, where applicable:

- Accession codes, unique identifiers, or web links for publicly available datasets
- A description of any restrictions on data availability
- For clinical datasets or third party data, please ensure that the statement adheres to our policy

Raw data are available from the authors upon reasonable request. The previously published single cell RNA-Seq data displayed in Extended Data Figure 1 are available at:
https://kleintools.hms.harvard.edu/tools/springViewer_1_6_dev.html?datasets/Sharma2019/all

Field-specific reporting

Please select the one below that is the best fit for your research. If you are not sure, read the appropriate sections before making your selection.

☒ Life sciences ☐ Behavioural & social sciences ☐ Ecological, evolutionary & environmental sciences

For a reference copy of the document with all sections, see nature.com/documents/nr-reporting-summary-flat.pdf

Life sciences study design

All studies must disclose on these points even when the disclosure is negative.

Sample size	No analyses were performed in advance to pre-determine sample size. Sample sizes were based on similar studies in the literature (Ranade et al. 2014, Pan et al. 2019).
Data exclusions	In the calcium imaging dataset in Figure 2, 57 individual neurons with compound addition artifacts (large spikes in the calcium imaging trace triggered upon drug addition) were excluded from AUC analysis but were still used for peak normalized ratio analysis. No other data were excluded.
Replication	All attempts at replication were successful. All experiments were repeated more than once as indicated in the figure legends except for Extended Data Figures 7r, 8a, and 10 and N is indicated for those experiments in the figure legends. For those experiments repeated only once, it is stated as such in the figure legend.
Randomization	No randomization was employed. Mice were arbitrarily assigned to treatment and vehicle groups for the GsMTx4 and Yoda1 experiments, as they were of identical age, genotype, and sex, so no randomization was possible. For all other behavior experiments, entire cohorts/litters of mice were tested at once by a blinded experimenter so no allocation or randomization was needed nor possible. Mice were arbitrarily assigned behavioral chamber numbers by the blinded experimenter. For electrophysiology and calcium imaging, a single coverslip or chamber of cells from each genotype/condition was tested in alternating order with the opposing genotype or condition (e.g. siRNA or drug treatment) so that genotypes/conditions were assessed in parallel. For all other experiments, no randomization was needed nor possible as there were no conditions to compare between.
Blinding	For all behavioral experiments, the experimenter and scorer/analyzer was blinded whenever possible to both treatment (when 2 or more treatments were applied) and/or genotype (when 2 or more genotypes were tested). For calcium imaging, data were analyzed offline using automated routines and so blinding was not necessary. For electrophysiology, experiments were conducted as previously published without blinding. For all other experiments, there were no comparisons so blinding was unnecessary.

Reporting for specific materials, systems and methods

We require information from authors about some types of materials, experimental systems and methods used in many studies. Here, indicate whether each material, system or method listed is relevant to your study. If you are not sure if a list item applies to your research, read the appropriate section before selecting a response.

Materials & experimental systems

n/a	Involved in the study	
☐	☒	Antibodies
☒	☐	Eukaryotic cell lines
☒	☐	Palaeontology and archaeology
☐	☒	Animals and other organisms
☐	☒	Human research participants
☒	☐	Clinical data
☒	☐	Dual use research of concern

Methods

n/a	Involved in the study	
☒	☐	ChIP-seq
☒	☐	Flow cytometry
☒	☐	MRI-based neuroimaging

Antibodies

Antibodies used	Primary antibodies: Rabbit anti-RFP (Rockland #600-401-379), Rat anti-PECAM1 (Sigma CBL1337-I), Chicken anti-NefH (Abcam #ab4680), Rabbit anti-CGRP (Immunostar #24112).<br>Secondary antibodies: Goat anti-Rabbit AlexaFluor 594 (Life Technologies #A11037), Donkey anti-Rat AlexaFluor 488 (Jackson #712-546-153), Donkey anti-Chicken AlexaFluor 647 (Jackson #703-605-155), Goat anti-rabbit AlexaFluor 488 (Thermo Fisher #A32731).
Validation	All antibodies used in the manuscript are previously published for use with mouse tissues with relevant citations available through the manufacturer's product page. We performed "no primary" controls for all experiments to validate lack of staining with our secondary antibodies. The primary antibodies were validated as follows.<br>Rabbit anti-RFP: (from manufacturer's website) "Assay by immunoelectrophoresis resulted in a single precipitin arc against anti-Rabbit Serum and purified and partially purified Red Fluorescent Protein (Discosoma). No reaction was observed against Human,

Mouse or Rat serum proteins." Citation: Dahl SL et al. Fate-mapping of erythropoietin-producing cells in mouse models of hypoxaemia and renal tissue remodelling reveals repeated recruitment and persistent functionality. (2022)
Rat anti-PECAM1: (from manufacturer's website) "A representative lot localized PECAM-1 immunoreactivity by fluorescent immunohistochemistry staining of murine embryo cryosections (Baldwin, H.S., et al. (1994). Development. 120(9):2539-2553)."
Chicken anti-NefH: (from manufacturer's website) "Our Abpromise guarantee covers the use of ab4680 in the following tested applications. ICC, IHC, WB." Citation: Hill RZ et al. The signaling lipid sphingosine 1-phosphate regulates mechanical pain. Elife (2018).
Rabbit anti-CGRP: (from manufacturer's website) "The antibody has a proven and strong Biotin-Streptadvidin/HRP staining at a 1/2000-1/4000 dilution in rat amygdala, and spinal cord. The specificity of the antiserum was evaluated by soluble pre-adsorption with the peptides in question at a final concentration of 10-5M. CGRP immunolabeling was completely abolished by pre-adsorption with rat α -CGRP and partially eliminated by pre-adsorption with rat CGRP. Pre-adsorption with the following peptides resulted in no loss of immunostaining: rat amylin, rat adrenomedulin, calcitonin, neurotensin, somatostatin, substance P, leucine enkephalin, methionine enkephalin, VIP, CCK-8, vasopressin and neuropeptide Y." Citation: Walsh CM et al. Neutrophils promote CXCR3-dependent itch in the development of atopic dermatitis. Elife (2019).

Animals and other organisms

Policy information about studies involving animals; ARRIVE guidelines recommended for reporting animal research

Laboratory animals	All experiments were performed under the policies and recommendations of the International Association for the Study of Pain and approved by the Scripps Research Animal Care and Use Committee. Mice were kept in standard housing with 12 h light/dark cycle set with lights on from 6am to 6pm, with room temperature kept around 22C, and humidity between 30-80% (not controlled). Mice were kept on pelleted paper bedding and provided with paper square nestlets and PVC pipe enrichment with ad libitum access to food and water. Age-matched littermate animals were used for all in vivo experiments. For all in vivo experiments except for Fig. 3h-iand Fig. 4d-i, which used only male mice, male and female mice were used and pooled. Mouse ages ranged from 2-6 months for behavioral studies, and 1.5-4 months for electrophysiology, calcium imaging, IHC, and smFISH. The homozygous Piezo1TdTomato animals were previously described22 and were maintained in the laboratory (B6;129-Piezo1tm1.1Apat/J; Jackson Laboratories #029214). The HM3dGqfl/fl; MrgprdCreERT2+/- animals were generated by crossing commercially available HM3dGqfl/fl mice (B6N;129-Tg(CAG-CHRM3*,-mCitrine)1Ute/J; Jackson Laboratories #026220) with MrgprdCreERT2+/- mice (Mrgprdtm1.1(cre/ERT2)Wql; Jackson Laboratories #031286), and intercrossing the progeny to obtain the desired genotypes. Recombination was achieved with once-daily intraperitoneal injection of 75 mg/kg body weight tamoxifen (Sigma) dissolved in 0.22 μm sterile-filtered corn oil delivered to both experimental and control animals over 5 consecutive days. The Ai9fl/fl; SstCre+/- animals were generated by crossing commercially available Ai9fl/fl female mice (B6.Cg-Gt(ROSA)26Sortm9(CAG-tdTomato)Hze/J; Jackson Laboratories #007909) with Ai9fl/fl; SstCre+/+ males (B6J.Cg-Ssttm2.1(cre)Zjh/MwarJ; Jackson Laboratories #028864). Visibly pink/red animals were not used for experiments, as some germline recombination was observed. Piezo1fl/fl; SstCre+/- animals were also generated from this line. Piezo1fl/fl; PirtCre+/- animals were generated by crossing Piezo1fl/fl female mice (Piezo1tm2.1Apat/J; Jackson Laboratories #029213) with PirtCre+/- males (Pirttm3.1(cre)Xzd, gift from X. Dong, Johns Hopkins University), and then crossing the Piezo1fl/+; PirtCre+/- male offspring with Piezo1fl/fl or Piezo1fl/+; Ai9fl/+ or Ai9+/+ female mice to generate homozygous knockouts, heterozygous animals, and PirtCre-/- control animals, some of which carried the Ai9fl/+ allele. The PIEZO1GOF mouse line ubiquitously carries the nucleotide change c.GG7742-7743AC and has been previously described26. Experimental PIEZO1GOF mice were generated from heterozygous matings. These above strains were maintained on a C57BL6/J background when not intercrossed to generate desired genotypes, except for Piezo1TdTomato and MrgprdCreERT2+/- which were maintained as inbred stocks. C57BL6/J wild-type male mice used in Figures 3h-iand 4d-I were purchased from the Scripps Research Department of Animal Resources rodent breeding colony. PCR genotyping from tail snip DNA samples was performed in-house using guidelines from Jackson Laboratory. All mice except for those purchased C57BL6/J mice received metal identification tags (National Band & Tag, 1005-1) on the right ear when they were between 18-30 days old. Upon weaning between 21-30 days of age, mice were co-housed in groups of 2-5 littermates of the same sex.
Wild animals	No wild animals were used in the study.
Field-collected samples	No field-collected samples were used in the study.
Ethics oversight	All experiments were approved by the Scripps Research Animal Care and Use Committee under protocol number 08-0136.

Note that full information on the approval of the study protocol must also be provided in the manuscript.

Human research participants

Policy information about studies involving human research participants

Population characteristics	The human DRG tissue was from N=1 female donor of Hispanic ethnicity aged 45 (AnaBios donor number 210204DHA). The donor had a history of cardiac disease, alcohol, tobacco, and drug use and had no history of neurological disease. The cause of death was listed as anoxia/cardiovascular. The donor demographic form was anonymized according to HIPAA regulations.
Recruitment	Informed consent for human tissue sources were obtained by AnaBios, Inc. (San Diego, CA). The consent for research was confirmed by the AnaBios coordinator and was signed and dated 5/20/2021. All human tissue used for this study were nontransplantable and ethically obtained by informed legal consent (first person or next-of-kin) from cadaveric organ donors in the United States (US).
Ethics oversight	We used donated human DRG tissue isolated and prepared by AnaBios, a company based in the United States of America. The following was provided from AnaBios: "Our recovery protocols and in vitro experimentation were preapproved by IRBs (Institutional Review Boards) at transplant centers within the US OPTN (Organ Procurement Transplant Network). Furthermore, all transfers of the donor tissues are fully traceable and periodically reviewed by US Federal authorities. All

human DRGs used for the study were obtained by legal consent from organ donors in the United States (US). AnaBios Corporation’s procurement network includes only US based Organ Procurement Organizations and Hospitals. Policies for donor screening and consent are the ones established by the United Network for Organ Sharing (UNOS). Organizations supplying human tissues to ANABIOS follow the standards and procedures established by the US Centers for Disease Control (CDC) and are inspected biannually by the United States Department of Health and Human Services (DHHS). Tissue distribution is governed by internal Institutional Review Boards (IRB) procedures and compliance with the Health Insurance Portability and Accountability Act (HIPAA) regulations regarding patient privacy. All transfers of donor organs to AnaBios are fully traceable and periodically reviewed by US Federal authorities. Donor tissues from males and females were harvested using AnaBios’ proprietary surgical techniques and tools and were shipped to AnaBios via dedicated couriers. Upon arriving at AnaBios, each DRG was assigned a unique identifier number that was reproduced on all relevant medical history files, data entry forms and electronic records. "

Note that full information on the approval of the study protocol must also be provided in the manuscript.
